# A multi-parent recombinant inbred line population of *C. elegans* allows identification of novel QTLs for complex life history traits

**DOI:** 10.1186/s12915-019-0642-8

**Published:** 2019-03-12

**Authors:** Basten L. Snoek, Rita J. M. Volkers, Harm Nijveen, Carola Petersen, Philipp Dirksen, Mark G. Sterken, Rania Nakad, Joost A. G. Riksen, Philip Rosenstiel, Jana J. Stastna, Bart P. Braeckman, Simon C. Harvey, Hinrich Schulenburg, Jan E. Kammenga

**Affiliations:** 1 Laboratory of Nematology, Wageningen University, Droevendaalsesteeg 1, NL-6708 PB Wageningen, The Netherlands; 2 Theoretical Biology and Bioinformatics, Utrecht University, Padualaan 8, 3584 CH Utrecht, The Netherlands; 3 Bioinformatics Group, Wageningen University, Droevendaalsesteeg 1, NL-6708 PB Wageningen, The Netherlands; 4 Biomolecular Research Group, School of Human and Life Sciences, Canterbury Christ Church University, North Holmes Road, Canterbury, CT1 1QU UK; 5 Department of Biology, Ghent University, K. L. Ledeganckstraat 35, B-9000 Ghent, Belgium; 6 Zoological Institute, University of Kiel, 24098 Kiel, Germany; 7 Institute for Clinical Molecular Biology, University of Kiel, 24098 Kiel, Germany; 8 Max Planck Institute for Evolutionary Biology, August-Thienemann-Str. 2, 24306 Plön, Germany

**Keywords:** Multi-parent RILs, *C. elegans*, QTL, Life-history, Natural variation, Genetic map

## Abstract

**Background:**

The nematode *Caenorhabditis elegans* has been extensively used to explore the relationships between complex traits, genotypes, and environments. Complex traits can vary across different genotypes of a species, and the genetic regulators of trait variation can be mapped on the genome using quantitative trait locus (QTL) analysis of recombinant inbred lines (RILs) derived from genetically and phenotypically divergent parents. Most RILs have been derived from crossing two parents from globally distant locations. However, the genetic diversity between local *C. elegans* populations can be as diverse as between global populations and could thus provide means of identifying genetic variation associated with complex traits relevant on a broader scale.

**Results:**

To investigate the effect of local genetic variation on heritable traits, we developed a new RIL population derived from 4 parental wild isolates collected from 2 closely located sites in France: Orsay and Santeuil. We crossed these 4 genetically diverse parental isolates to generate a population of 200 multi-parental RILs and used RNA-seq to obtain sequence polymorphisms identifying almost 9000 SNPs variable between the 4 genotypes with an average spacing of 11 kb, doubling the mapping resolution relative to currently available RIL panels for many loci. The SNPs were used to construct a genetic map to facilitate QTL analysis. We measured life history traits such as lifespan, stress resistance, developmental speed, and population growth in different environments, and found substantial variation for most traits. We detected multiple QTLs for most traits, including novel QTLs not found in previous QTL analysis, including those for lifespan and pathogen responses. This shows that recombining genetic variation across *C. elegans* populations that are in geographical close proximity provides ample variation for QTL mapping.

**Conclusion:**

Taken together, we show that using more parents than the classical two parental genotypes to construct a RIL population facilitates the detection of QTLs and that the use of wild isolates facilitates the detection of QTLs. The use of multi-parent RIL populations can further enhance our understanding of local adaptation and life history trade-offs.

**Electronic supplementary material:**

The online version of this article (10.1186/s12915-019-0642-8) contains supplementary material, which is available to authorized users.

## Background

Determining genotype-phenotype relationships is the heart of genetics. Understanding how the relationships between traits, genotypes, and environments are controlled is also crucial to find traits relevant to the evolved context of the species [1–7]. The identification and characterization of allelic variants associated with complex traits have been a major challenge in plant and animal breeding as well as disease genetics. Many complex traits vary in a continuous way across different genotypes of a species. It is this phenotypic variation that can be mapped to the genome using quantitative trait locus (QTL) analysis. Standard QTL mapping for many different species is based on recombinant inbred lines (RILs) derived from a cross between two genetically and phenotypically divergent parents. One of the many species that has extensively been used for exploring the genetics of complex traits is the free-living, bacterivorous nematode *Caenorhabditis elegans*, reviewed in [8, 9].

*C. elegans* is found in most temperate regions in the world [10–12]. In the wild, this nematode is mostly found on rotten (plant) material and is also found associated with snails, slugs, and other invertebrates [11, 12]. Populations of this opportunistic species can proliferate very fast (boom-and-burst). Generally, only a single *C. elegans* nematode reaches a new food source (e.g., rotting hogweed stem) and colonizes this resource. When food gets scarce, population growth halts and juveniles enter the long-lived dauer stage. In this stage, *C. elegans* nematodes display nictation behavior, allowing association with an invertebrate host [11, 13, 14]. Only a few juveniles of the colony will reach the next food source. Mostly, only a single genotype is found to dominate a food source, yet in some cases, multiple genotypes are found to co-occur [15]. Generally, a single genotype is dominant in an area but can be replaced over time [15, 16].

In nature, *C. elegans* reproduces primarily through self-fertilization although outcrossing with males occurs occasionally [15–18]. Individual worms typically produce up to 350 offspring when self-fertilizing, and the generation time of *C. elegans* is 3.5 days (at 20 °C). Outcrossing could be hampered due to genetic incompatibilities [19, 20] and possibly outbreeding depression [21]. Both effects could account for the low frequency of heterozygous *C. elegans* nematodes in nature and the dominance of a single genotype in most areas [15–18]. At the same time, a certain level of genetic diversity, at least at single loci, may be maintained by balancing selection, which has been repeatedly identified in natural *C. elegans* populations, for example, in relation to genetic mating incompatibility [19], virus resistance [22], and foraging behavior [23]. Both outbreeding depression and balancing selection may favor epistatic interactions among loci, which jointly influence the traits under selection, further shaping the genetic composition of natural worm populations. Moreover, *C. elegans* population genetics are also shaped by the boom-and-bust lifestyle of this nematode species, which is particularly common in ephemeral habitats, where it is subject to frequent extinction and re-colonization events (reviewed in [11, 12]). As a consequence of these different dynamics and selective forces, genetic diversity between *C. elegans* populations on a local scale can be almost as diverse as on a global scale, with genetically distinct populations occurring within a few kilometers distance [10, 17, 18, 24, 25]. The analysis of RILs, which are constructed from natural isolates, may then help us understand at least some of the involved dynamics, especially as to the traits under selection and the underlying genetic architecture. Furthermore, the generation of RILs will break up strong interacting loci, possibly revealing the underlying genetic architecture of complex traits [26, 27], as exemplified by the dissection of genetic incompatibility loci [19, 28]. Creating a population of RILs from a cross between multiple locally isolated *C. elegans* haplotypes and thus generation of multi-allelic genetic mosaics enables the exploration of the link between lifecycle dynamics, genetic variation, and evolutionary processes.

Most inbred mapping populations of *C. elegans* were derived from two globally distant locations, namely Bristol, UK (N2 strain) and Hawaii (CB4856 strain) [9, 29–32]. These Bristol-Hawaii RIL populations have been very valuable for studying the genetic architecture of complex traits [33–45] and the identification of the underlying genes [23, 46–56], even though other genotypes have been used for the construction of RIL populations, e.g., crosses between N2 and BO [57], N2 and DR1350 [43], N2 and LSJ1 [58], JU605 and JU606 [59], MT2124 and CB4856 [60], and JU1395 and MY10 [61]. Typically, these additional RILs have only been used to address a specific research question, and thus, their suitability to map QTLs for different types of traits is unclear compared to the work on the Bristol-Hawaii populations. Although other types of crossing strategies involving multiple lines [62] or panels of wild isolates have been reported, most of the work has been done on the Bristol-Hawaii-derived RILs which only captures a subset of the phenotypic and genetic diversity present in *C. elegans.*

The inclusion of more than 2 parental sources of genetic variation and alleles captures more genetic variation and allows for more precise mapping and identification of potential regulatory variants of complex traits [63]. An alternative to the conventional 2 parental genetic mapping strategies is the development of Multiparent Advanced Generation Inter-Cross (MAGIC) lines. The first of such populations was developed for *Arabidopsis thaliana* consisting of 527 RILs developed from 19 different parental accessions [64]. Since then, many more MAGIC populations have been developed for a range of species [65]. Recently, a *C. elegans* multi-parental RIL population originating from 16 wild types that underwent almost 200 generations of experimental evolution [62] was characterized [26]. This RIL panel comprised of 507 strains covers about 22% of single nucleotide polymorphisms (SNPs) known to segregate in natural populations [26, 62].

Here, we report the construction and analysis of a multi-parental (4) recombinant inbred line (mpRIL) population for *C. elegans*. The 200 mpRILs are derived from an advanced cross between 4 wild types: JU1511 and JU1941 isolated from Orsay (France) and JU1926 and JU1931 isolated from Santeuil (France) (kindly provided by MA Félix, Paris, France; [25]). The RILs were SNP genotyped based on RNA-seq data. We used the SNP-genotyped lines for mapping QTLs for the following *C. elegans* phenotypes: length, width, length to width ratio, volume, lifespan, lifespan during dietary restriction, heat-shock survival, oxidative stress, occurrence of males, the developmental speed on the food sources of *Escherichia coli* OP50 and naturally *C. elegans*-associated bacteria *Erwinia rhapontici* [25, 66], and population growth on *E. coli* OP50 and naturally *C. elegans*-associated bacteria *Erwinia rhapontici*, *Sphingomonas* sp., non-pathogenic *Bacillus thuringiensis* strain DSM-350E, and the pathogenic *Bacillus thuringiensis* strain NRRL B-18247 [25]. We aimed to measure a range of traits under different bacterial food conditions and abiotic conditions that, to a certain extent, reflect natural conditions [12, 25, 67]. For all these traits, heritable variation and QTLs were found. Here, we present a new multi-parental recombinant inbred line population and show the distribution of genetic variation, recombination, trait variation, and identified quantitative trait loci and show the effects of local genetic variation on phenotypic traits.

## Results

### Developing a *C. elegans* multi-parental recombinant inbred line population

**Fig. 1:**
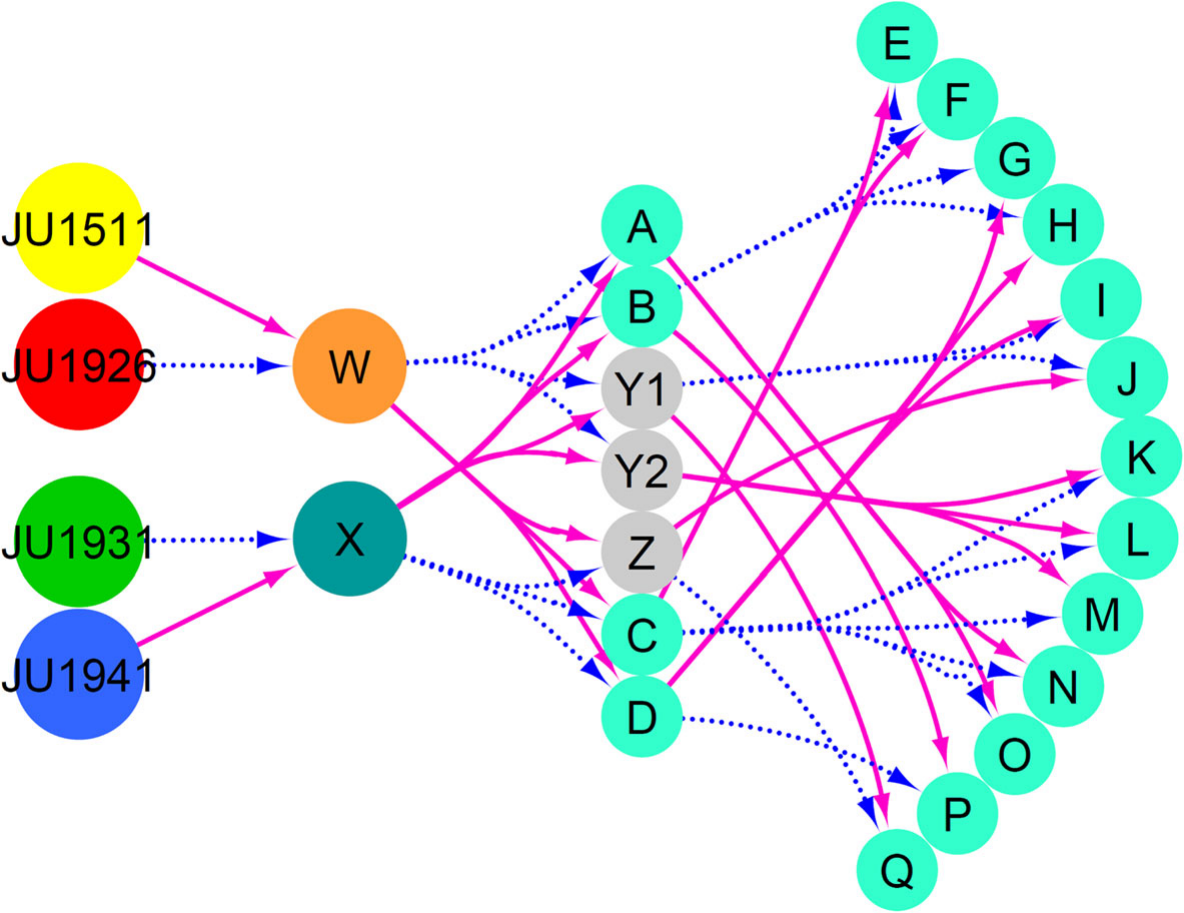
Crossing scheme used to make the four parental mpRIL population. Different populations of worms are shown in the colored circles. Magenta solid lines indicate the hermaphrodite parental lines, and the dashed blue lines show the male parental lines. A JU1511 hermaphrodite (yellow) was crossed with a JU1926 male (red) to create F1 population W. A JU1941 hermaphrodite (blue) was crossed with a JU1931 male (green) to create F1 population X. Individuals from populations W and X were reciprocally crossed to create seven separate populations. In this way, we obtained different populations of genotypes with mixed genetic background from the four parental lines. Individuals from the seven separate populations (A, B, Y1, Y2, Z, C, and D) where further intercrossed to obtain extra recombinations mainly to break up the X-chromosome, which lacks recombination in the male. These populations are labeled E to Q. From these populations (light blue), individuals were taken for inbreeding via self-fertilization for six generations to create mpRILs. For details, see also Additional file 1: Table S1 and Fig. 3

To allow the 4 parental genomes (JU1511, JU1941, JU1926, and JU1931) [25] to recombine, we set up a crossing scheme in which 2 pairs of wild isolates were crossed and both the obtained F1 populations were reciprocally inter-crossed (Fig. 1; Additional file 1: Table S1). To enable crossovers on chromosome X and to generate extra crossovers, the heterozygous F2 obtained from these initial crosses were further inter-crossed. To create homozygous genotypes, single worms were selected from the F2 as well as from the F2 inter-cross for 6 generations of single-worm inbreeding. From these 383 lines, a population of 200 different multi-parental recombinant inbred lines (mpRILs) was randomly picked for mRNA sequencing to obtain the genetic variation in the coding sequence.

### Polymorphisms are not distributed equally

**Fig. 2:**
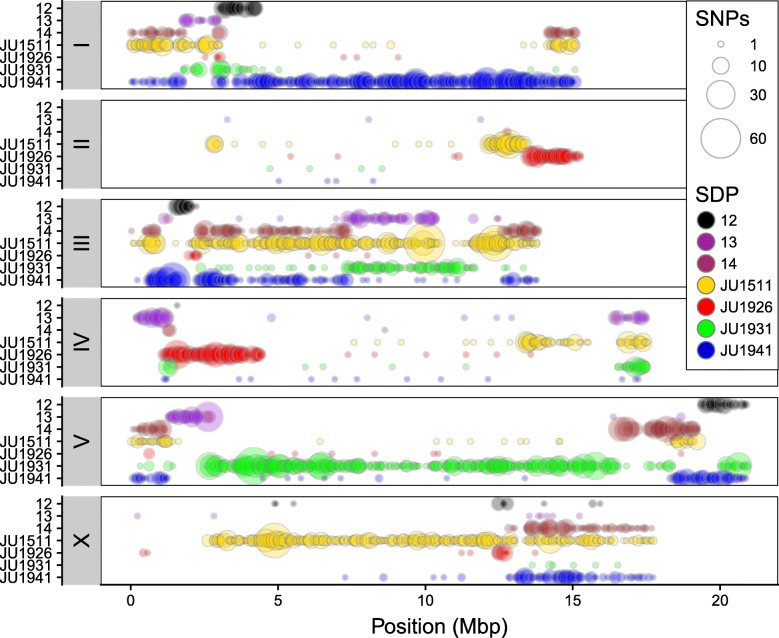
Genome-wide SNP distribution in the four parental genotypes. Circle size shows the number of SNPs within 50 kb bin. Colors indicate SNP distribution patterns (SDP) as shown in the legend. These are SDP 12—difference between pair JU1511/JU1926 and pair JU1931/JU1941; SDP 13—difference between pair JU1511/JU1931 and pair JU1926/JU1941; and SDP 14—difference between pair JU1511/JU1941 and pair JU1926/JU1931

For genotyping of the 200 mpRILs, we used the single nucleotide polymorphisms (SNPs) obtained from RNA-seq. We detected 8964 SNPs diverging in the coding sequences between the parental lines. The distribution of these SNPs over the genotypes can be grouped into 7 specific *SNP distribution patterns* (SDP): 4 patterns represent the 4 parental strains; 3 patterns are shared between 2 parental strains versus the other 2 parental strains. These are SDP 12—difference between pair JU1511/JU1926 and pair JU1931/JU1941; SDP 13—difference between pair JU1511/JU1931 and pair JU1926/JU1941; and SDP 14—difference between pair JU1511/JU1941 and pair JU1926/JU1931. Importantly, SDP 14 therefore represents SNPs diverging between the 2 isolation sites, and hence, these polymorphisms may be informative of local adaptation. The SNP distribution differed between parents, and the SNPs were unequally distributed across the genome (Table 1; Fig. 2; Additional file 1: Table S2). For example, chromosomes I, III, and V were more polymorphic in their coding sequences compared to chromosomes II, IV, and X. Overall, chromosome II was the least polymorphic and chromosome III was the most polymorphic. Furthermore, we found regions where multiple SDP overlap (left arm of chromosomes I, IV, and V; right arm of chromosomes I, IV, V, and X, and all of chromosome III; Fig. 2), which can potentially be used to reduce the number of candidate causal SNPs.

**Table 1:** SNP distribution patterns (SDP). Distribution of SNPs and alleles in total and per chromosomes I to X for each SDP. Strain names and place of isolation are give in the column headers. At SDP 14, O vs S shows those SNPs different between the two strains isolated from Orsay and the two strains isolated from Santeuil

	Total	JU1511, Orsay	JU1926, Santeuil	JU1931, Santeuil	JU1941, Orsay	SDP 12	SDP 13	SDP 14, O vs S
Total	8964	2628	748	1651	2137	313	842	518
I	1916	288	10	75	1250	131	109	41
II	503	254	229	4	4	0	2	3
III	2231	979	16	217	476	72	281	158
IV	930	178	447	91	14	1	12	162
V	2152	104	11	1257	214	88	303	144
X	1232	825	35	7	179	21	135	10

Most SNPs were strain specific or diverging between strains from the two isolation sites. For example, unique SNPs on chromosome I were mostly specific for JU1941 alleles, while those on chromosome V were mostly specific for JU1931. For JU1511, unique SNPs were found on all chromosomes whereas the other parental genotypes have chromosomes almost completely lacking unique SNPs. Moreover, the genotypes from Orsay (JU1511 and JU1941) had more (> 2000) strain-specific SNPs compared to those from Santeuil (JU1926 and JU1931). Almost 1700 SNPs were found in Orsay vs. Santeuil genotypes, whereas only 518 (~ 30%) were shared between genotypes from the same geographical location.

Across all chromosomes, both the left and right arms of the chromosomes (except for chromosome III) were more polymorphic compared to the center of the chromosomes, a result matching that seen in previous work on *C. elegans* wild isolates [10, 29, 68]. Specific regions were very polymorphic between the four parental lines, such as the left arm of chromosome I, all of chromosome III, both arms of chromosome V, and the right arm of chromosome X. Long stretches of relative low SNP variation could also be observed, such as large parts of chromosome II, middle part of chromosome IV, and left arm of chromosome X. For the majority of the genome, at least one parental genotype could be uniquely identified by individual SNPs. Overall, we concluded that SNPs in coding regions of *C. elegans* were unequally distributed over the genome and among genotypes and that the chromosome arms were more polymorphic than the chromosome centers.

### Cross-specific recombination in the mpRILs

**Fig. 3:**
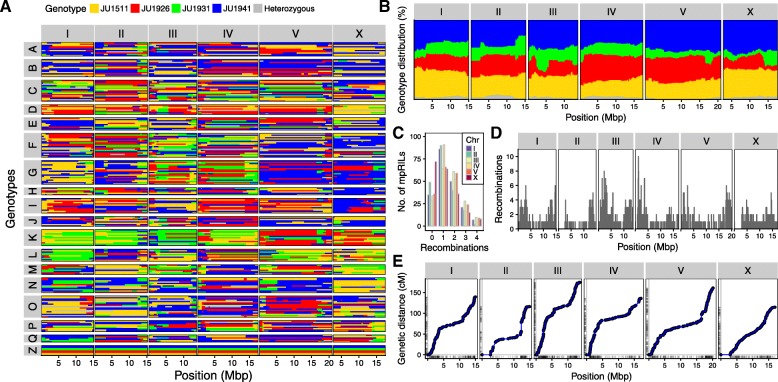
Parental background of the multi parental recombinant inbred lines and recombination and allelic distribution per chromosome. **a** Colors indicate the parental background per genetic segment (*x*-axis) per RIL (*y*-axis) as estimated from the SNP distribution patterns. Chromosomes are in separate panels on the *x*-axis. mpRILs are grouped according to their cross history. The parental lines are shown in group Z. **b** Genome-wide distribution of parental alleles. Colors indicate the percentage of parental occurrence (*y*-axis) per genetic segment (*x*-axis) as estimated from the SNP distribution patterns. Chromosomes are in separate panels on the *x*-axis. **c** Recombination per chromosome. Chromosomes show on the *x*-axis. Numbers of recombinations per RIL are shown on the *y*-axis. **d** Recombination frequency per chromosome. **e** Genomic distance (*x*) vs genetic distance (*y*), the rug lines (small lines on the axis) indicate the marker positions

The genetic map shows a highly variable frequency of recombination and introgression sizes (Fig. 3a; Table 2; Additional file 1: Tables S3 and S4). In total, 1683 recombination events were found in the mpRIL population, with a genome-wide allelic presence of the 4 parental lines (Fig. 3b, Additional file 2: Figure S1). Up to 4 recombination events per mpRIL per chromosome were found, where 1 or 2 recombination events per chromosome was most common (Fig. 3c). The average number of crossovers per mpRIL was 8.5 across all chromosomes and 1.5 per chromosome. Most recombination events were found on chromosome III (348). As expected, due to the lack of recombination of chromosome X in males, the fewest recombination events were found on chromosome X (233) (Fig. 3d). Moreover, for chromosome X, almost 40% of the mpRILs showed no recombination. The recombination rate was on average 1 per 17 Mb, with a genome-wide mean introgression size of 5.0 Mbp (median 3.1 Mbp). We observed a suppression of recombination across the centers of the chromosome with higher recombination rates at the chromosome arms (Fig. 3e), consistent with previous work on *C. elegans* [10, 29, 69]. Considering the whole population, the genomic bins (loci) that could be individually investigated had a median size of 43 kbp. (Table 2). The effective recombination rate useful for QTL mapping becomes larger as multiple SDP could be recombined by a single recombination event (Additional file 3: Figure S2). Including the SDP increased the effective recombination rate to approximately 57 per Mbp and 5686 recombination events in total. This did not affect the mapping resolution (making the QTLs smaller), yet it did reduce the amount of potentially causal polymorphisms, and therefore, mapping in an SDP-dependent manner affected the number of polymorphisms under investigation when looking for the causal gene or SNP.

**Table 2:** Crossovers, introgression size and bin size in total and per chromosome

	Total	Average per mpRIL	No. of mpRILs without COs	No. of mpRILs with COs	Mean introgression size (Mbp)	Median introgression size (Mbp)	Median bin size (kbp)
Total	1683	8.5	1	198	5.0	3.1	43.1
I	277	1.4	35	164	4.9	3.1	45.4
II	232	1.2	49	150	4.4	3.6	44.3
III	348	1.7	29	170	4.2	2.9	26.2
IV	272	1.4	34	165	6.0	3.4	45.1
V	321	1.6	36	163	6.3	3.7	43.1
X	233	1.7	72	127	3.8	2.5	68.9

The allelic distribution was different between cross and inbreeding pools. The ratio of parental alleles shows a similar distribution across the chromosomes, except for chromosome II (Fig. 3b). Alleles from all four parents had a genome-wide representation, although JU1931 alleles occurred less frequently genome-wide and JU1926 alleles occurred relatively less frequently on chromosomes I, III, and X. The allelic distribution was dependent on the specific cross and inbreeding pool (Fig. 1 and Additional file 2: Figure S1; Additional file 1: Table S1). In each specific pool, the parental alleles display a cross-specific and chromosome-specific distribution, frequently showing an absence of one or a few allele types (Additional file 2: Figure S1). Taken together, the whole population of mpRILs captures the genetic variation of the parental strains from which they were derived, perturbed by recombination.

### Phenotypic variation and heritability

**Fig. 4:**
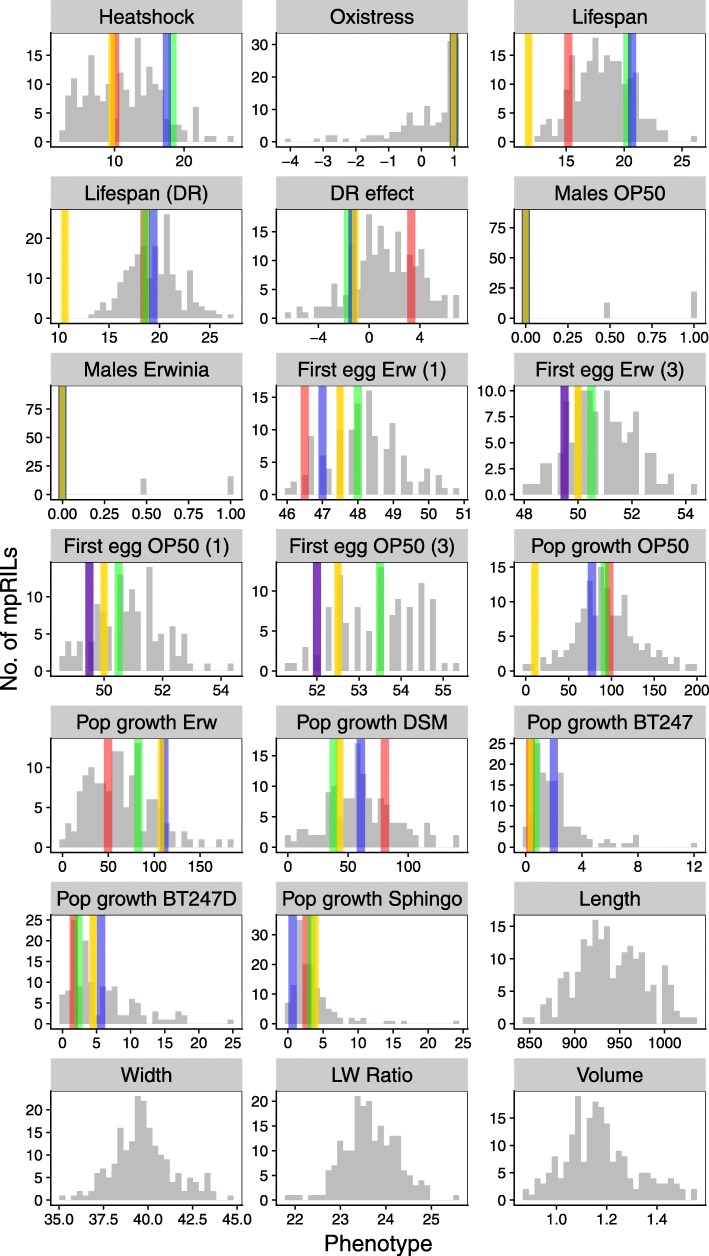
Phenotypic variation in the mpRILs. On the *y*-axis, the number of mpRILs is shown. The *x*-axis values depend on the trait. Heat shock is the average number of dead animals per 50. Oxidative stress indicates activity. Lifespan is the average lifespan on NGM in days. Lifespan (DR) is the average lifespan on DR medium in days. DR effect is the difference in the average lifespan between NGM and DR medium in days. Males OP50 and males *Erwinia* is the occurrence of males on plates (0 = none , 0.5 = 1 plate, 1 = 2 plates). First egg Erw (1) is the time in hours until the first egg (1–10) for populations grown on *Erwinia*. First egg Erw (3) is the time in hours until the first egg (> 100) for populations grown on *Erwinia*. First egg OP50 (1) is the time in hours until the first egg (1–10) for populations grown on OP50. First egg OP50 (3) is the time in hours until the first egg (> 100) for populations grown on OP50. Pop growth shows worms per 5 μl of culture. Length and width in nanometers, LW ratio is the ratio between the length and width; volume in nanoliters. The parental values for these traits are not shown yet were measured in a different batch in Volkers et al. 2013. Distribution in the mpRILs are shown in gray, and parental strains are shown JU1511 (yellow), JU1926 (red), JU1931 (green), and JU1941 (blue)

**Fig. 5:**
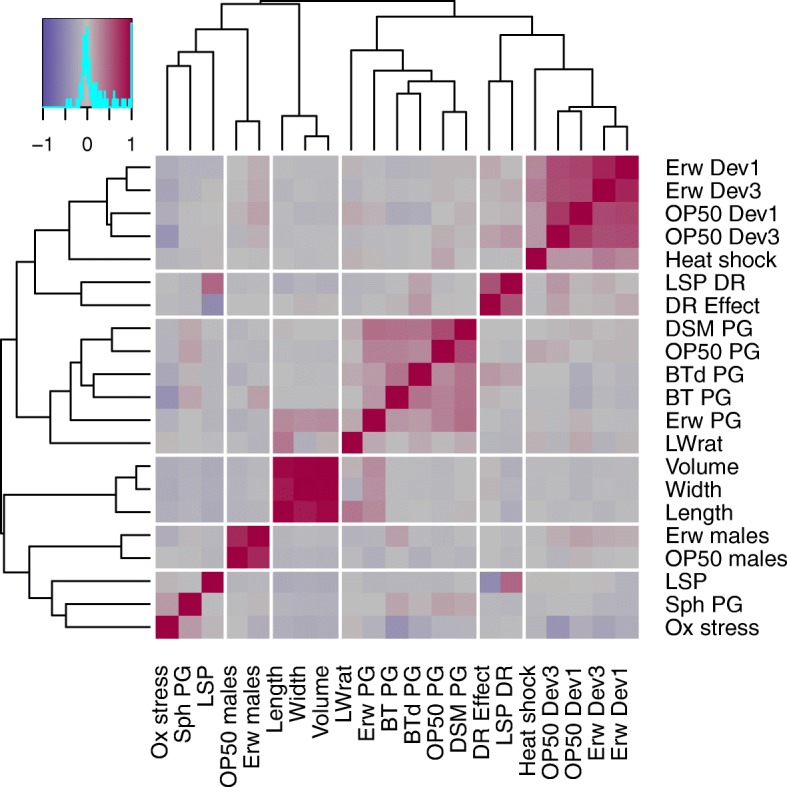
Correlations between traits in the mpRILs. Ewr Dev1 and Ewr Dev3 are the speed of development on *Erwinia* as measured by time until the first egg (1 = first egg, 3 = first > 100 eggs). OP50 Dev1 and OP50 Dev3 are the speed of development on OP50. Heat shock is the alive worms after 10 h of 35 °C. LSP DR is the lifespan on dietary restriction. DR Effect is the difference in lifespan between LSP DR and LSP. DSM PG, OP50 PG, BTd PG, BT PG, and Erw PG are the population growth on bacteria DSM, OP50, BT diluted, BT and *Erwinia*, respectively. LWrat is the length to width ratio. Volume is the animal volume. Width is the animal width. Length is the animal length. Erw males and OP50 males are the occurrence of males on *Erwinia* and OP50, respectively. LSP is the mean lifespan. Sph PG is the population growth on *Sphingmonas*. Ox stress is the oxidative stress survival

The studied phenotypes were chosen based on previous work on these *C. elegans* strains where we describe extensive phenotypic variation between the parental lines [25]. Moreover, we focused on a variety of traits that are generally relevant for the ecology and evolutionary fitness of *C. elegans* in nature. Even though the range of traits is non-exhaustive, they cover distinct ecologically important characteristics, including population growth, lifespan, speed of development, and stress resistance. We specifically considered some traits that are likely under high selection in nature, such as the response to natural food microbes, in case of *Erwinia* and *Sphingomonas*, isolated together with the parental strains [25] or pathogens, which we assessed using population growth as a meaningful proxy for worm fitness. For most traits, we observed substantial heritable phenotypic variation between mpRILs (Fig. 4; Additional file 1: Table S5 and Table S6). Correlation analysis (Fig. 5; Additional file 1: Table S7) across all phenotypic traits showed that the timing of the first eggs laid was highly correlated across different food conditions. This was also found for population growth, except growth on *Sphingomonas*. Body size and developmental phenotypes were also highly correlated, showing that these phenotypes were likely to share a similar genetic architecture.

We measured 21 phenotypes in the mpRILs of which 17 also in the parental wild types. Average lifespan was 18 days (range 13 to 26 days) and ~ 1 day longer under dietary restriction (DR) at 19 days (range 13 to 27 days). The overall effect of DR on lifespan was positive, but negative effects were observed for individual genotypes (approximately − 6 days to approximately + 7 days) as previously found in *C. elegans* [70] and in mice [71]. Heat shock (10 h at 35 °C) had a severe effect on the survival, on average ~ 11% (3 to 27) of the population survived 2 days after. Oxidative stress did not affect the average behavioral activity but did have an effect on individual genotypes (− 4 to − 1). Worms fed on *Erwinia* started laying their eggs earlier compared to worms fed on OP50. As previously found [25, 72], the average time of the first egg laid on *Erwinia* was shorter (mean ~ 51 h; range ~ 48 to ~ 54 h) than on OP50 (mean ~ 53 h; range ~ 51 to ~ 55 h). The occurrence of males was similar on both OP50 and *Erwinia*; for most mpRILs, no males were found, yet the genotypes that had males in the population did so on both OP50 and *Erwinia*. Population growth of the mpRILs differed strongly between worms fed with different bacteria as previously found between wild isolates [25]. On average, population growth was highest on OP50 (mean ~ 93 individuals/5 μl; range 0 to 197/5μl), on *Erwinia* (mean ~ 65/5 μl; range 0 to 183/5 μl), and DSM (mean ~ 61/5 μl; range 0 to 140/5 μl). Slow growth was observed on *Sphingomonas* (mean ~ 3/5 μl; range 0 to 24/5 μl) and on BT247 (mean ~ 2/5 μl; range 0 to 12/5 μl). The mpRILs are also variable in length (mean 945 μm; range 848 to 1135 μm), width (mean 40 μm; range 35 to 46 μm), length to width ratio (mean 24; range 22 to 26), and volume (mean 1.2 nl; range 0.9 to 1.9 nl).

The highest heritability (83%) was found for population growth on *Erwinia*, whereas the lowest (55%) was found on the most toxic concentration of BT247. Developmental speed for both OP50 and *Erwinia* showed high heritability (80%) with many mpRILs having phenotypes beyond the parental phenotypic values (Additional file 1: Table S6). Most mpRILs developed slower than the parents. Body size also showed high heritability (~ 80%), with a length to width ratio of ~ 70%. Population growth on the different bacteria showed variation in heritability, possibly linked to average growth rate on the specific bacteria. Transgression shows mpRILs beyond the parental phenotypes on both sides, yet for the growth on the BT247 strain, transgressive mpRILs mostly produce better growth than the parents.

Together, the results show that ample phenotypic variation of complex traits can be found between the mpRILs and that these phenotypic differences are heritable. Genetic variation across the mpRILS causal for these different functional differences is likely to have fitness effects.

### Quantitative trait loci

**Fig. 6:**
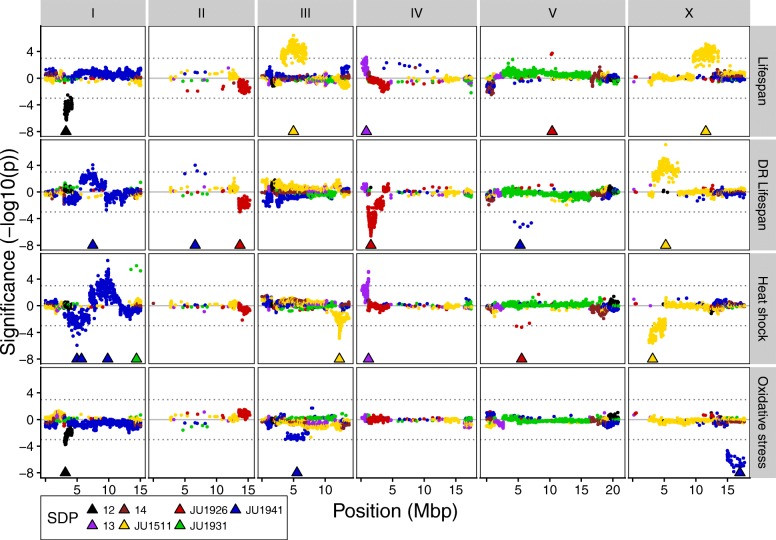
QTL profiles of the lifespan and stress phenotypes. Genomic position on the *x*-axis against the significance on the *y*-axis. Triangles show the position of the co-factors used in the final mapping model. Significance was multiplied by the sign of the allelic effect to show the effect direction. Colors show SNP distribution patterns (SDP). These are SDP 12—difference between pair JU1511/JU1926 and pair JU1931/JU1941; SDP 13—difference between pair JU1511/JU1931 and pair JU1926/JU1941; and SDP 14—difference between pair JU1511/JU1941 and pair JU1926/JU1931

**Fig. 7:**
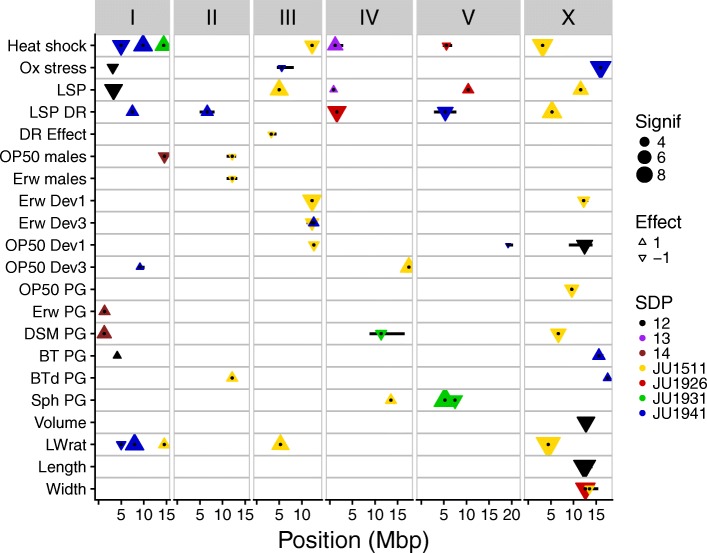
Genome-wide overview of QTLs. QTLs are shown by the triangles; triangles pointing upwards show a positive allelic effect, and triangles pointing downwards show a negative allelic effect. Size indicates significance in −log10(*p*); colors show SNP distribution patterns (SDP). These are SDP 12—difference between pair JU1511/JU1926 and pair JU1931/JU1941; SDP 13—difference between pair JU1511/JU1931 and pair JU1926/JU1941; and SDP 14—difference between pair JU1511/JU1941 and pair JU1926/JU1931. Black dots show the exact location of the peaks and the black horizontal bars the 2 −log10(*p*) drop QTL intervals

By applying quantitative trait locus (QTL) mapping using a forward co-factor selection approach, we identified the loci associated with variation in the measured phenotypic traits (Figs. 6 and 7; Additional file 1: Table S8; Additional file 4: Figure S3). The average QTL interval was 1.2 Mbp, median QTL interval was 0.88 Mbp, minimum QTL interval was 2.06 kbp, and maximum QTL interval was 7.7 Mbp. Most QTLs were found for the lifespan/stress traits (3–7 per trait), and together, these explained between 32 up to 41% of the total trait variation observed. Of the 21 lifespan/stress QTLs, most showed an allelic difference between JU1941 (7) or JU1511 (6) and the other 3 parental genotypes. The most significant QTL was found on chromosome X at ~ 16 Mbp for the effect of oxidative stress which explained 20% of the variation. For developmental speed, 2 to 3 QTLs were found per trait and together explained 24 to 31% of the total variation per trait. Again, most of the 9 QTLs found for all developmental speed traits showed an allelic difference between JU1941 (3) or JU1511 (5) and the rest. Of all these QTLs, a QTL found on chromosome III around 12.3 Mbp (for which the JU1511 allele shortens the developmental speed on *Erwinia* by almost 1 h) explained most variations (22%). Population growth traits showed between 1 and 3 QTLs, where the 3 QTLs for both *Sphingomonas* and DSM explained ~ 30% of the variation. The growth on other bacteria had less than 16% of the variation explained by the identified QTLs. The most significant QTL was found on chromosome V at 5.3 Mbp for the population growth on *Sphingomonas*, where the JU1931 allele increased population growth. For the body size traits, 1 to 2 QTLs were found explaining up to 16% of the phenotypic variation, with the exception of the length to width ratio for which 5 QTLs were found explaining 38% of the variation. Overall, we found that QTLs were mostly determined by JU1511-specific (21 QTLs) and JU1941-specific (14 QTLs) SNPs and relatively few by other alleles. Comparison of major effect QTL locations for the different traits identifies no clear evidence of trade-offs between traits measured in this study and only limited evidence for genomic regions affecting multiple traits simultaneously (Fig. 7).

## Discussion

We have developed a new *C. elegans* multi-parental recombinant inbred line population (mpRILs) derived from four wild-type strains capturing local genetic variation. This population of mpRILs complements existing RIL panels derived from two founders [29, 30, 43, 57, 59] and a multi-parent population derived from experimental evolution lines [26]. The four founding parental wild types originated from two different locations in France, Orsay (JU1511 and JU1941) and Santeuil (JU1926 and JU1931), collected and provided by Marie-Anne Félix and described in [25]. These strains were selected because of their genetic differences with each other. They also differed strongly compared to the two widely used strains Bristol N2 and Hawaii CB4856 [25]. Nevertheless, any small number of strains will only encompass a limited part of the total genetic variation in *C. elegans*. Yet, allele coverage will increase with an increasing number of RIL populations with different parental strains, thereby enhancing our overall ability to dissect the functional consequences and possible underlying evolutionary causes of global genetic variation.

Our mpRIL population is the first *C. elegans* population genotyped by SNPs in coding regions using RNA-seq. As RNA was isolated at a single time point (stage L4) during the life of the worm, these SNPs are limited to coding regions of genes that are expressed at that particular age. This limits the detection of SNPs to a temporally defined collection of expressed genes. There is a chance that this biases the detection of QTLs to SNPs of genes expressed at this point in the worm’s life. However, comparing the SNP distribution to genomic SNPs in *C. elegans*, we show that the SNPs based on RNA-seq are distributed according to expectation [10, 29]. Therefore, these SNPs are representative of the total genetic variation across the four strains. Hence, the QTLs detected are likely to be unbiased. Nonetheless, DNA sequencing would be beneficial to get better coverage in regions where coding SNPs are sparse and to detect the non-coding SNPs and other polymorphisms that can be potentially causal for observed phenotypic variation between the mpRILs.

Recombination frequencies detected based on RNA-seq are also not likely to be biased when compared to those from 2 parental RIL panels characterized by classical genetic markers or through genome sequencing. Yet, the average spacing between SNPs will be affected by RNA-seq compared to DNA-seq based. Our mpRIL population has a recombination frequency of 16.8 events per megabase pair and a mean introgression size of 5.0 Mbp. These 2 measures might be underestimated as the regions, where only non-coding variation exists between 2 or more lines recombination becomes impossible to detect. The previously described 2 parental RIAIL populations [29] showed a higher genome-wide recombination frequency of 36 per megabase pair, most likely due to the advanced inter-cross design that was used to generate the RIAILs [29]. Overall, we found approximately 9000 SNPs with an average spacing of 11 kb compared to 1454 nuclear SNP markers with a spacing of 61,160 bp in the RIAIL population. A spacing of 1 SNP per 20 kb was reported for a multi-parental experimental evolution (CeMEE) panel, derived from 16 parental strains in *C. elegans* [26]. As far as these results can be compared, they show that all these populations are in the same range regarding SNP density, with an overall increase in SNP density for our mpRIL population.

Heritability values (50–80%) are relatively high for some traits compared to those previously reported (~ 20%) [73] yet similar to heritability values found for related fitness traits across other species. Moreover, the values reported in our study are in line with those reported by Noble et al. [26]. It should be noted that heritability is a characteristic of a trait in a population and not of an individual trait in general. Therefore, heritability values are dependent on both the environmental and genetic circumstances of a population. Future studies with different RIL panels will help to understand to what extent heritability for specific traits can vary between populations of a species and whether and why there may be upper or lower boundaries for such measures. The range of inbred populations currently becoming available in *C. elegans* may be ideal to dissect variation in heritability in more detail.

We have investigated the genetic architecture of a range of complex traits and identified single—and usually multiple—QTLs underlying the observed variation. Overall, QTLs with a JU1931 (4), JU1926 (4), or SDP 13 (2) allelic effect were found least frequently. QTLs with a JU1511 (21) and JU1941 (14) allelic effect were most abundant. This differential pattern of allelic effects highlights another advantage of using a mpRIL population over classical two parental RIL populations. Around 80% of the QTLs would have been found when just JU1511 and JU1941 had been used as parental strains for a RIL population. In contrast, only 30% of the QTLs would have been found if JU1926 and JU1931 served as parental strains. Furthermore, in the regions where multiple SDP overlap (left arm of chromosomes I, IV, and V; right arm of chromosomes I, IV, V, and X; and all of chromosome III), the number of potential causal SNPs can be reduced, as each QTL is mapped on a specific SDP. Based on the coding SNPs, the estimated reduction in potential causal SNPs was found to be 30–99% of the total SNPs on the QTL (Additional file 1: Table S8). It is tempting to speculate about local adaptation and niche formation as causal for the fact that most QTLs have a specific allelic effect from one of the Orsay strains (JU1511 or JU1941), yet it could be as simple as those lines having the most strain-specific (not shared) SNPs over the largest parts of the genome leading to more specific QTL effects.

We compared the detected QTLs in the mpRILs to those reported from other studies, revealing the identification of novel QTLs. For example, for variation in lifespan, in a two-parent N2 x CB4856 IL population, QTLs were detected on chromosomes II and IV [20] and in another study on chromosomes I, II, III, IV, and X [32] while in the current study, QTLs for the same trait were found on chromosomes I, III, IV, V, and X (Fig. 7)—without any apparent overlap. For lifespan under DR, we identified QTLs on chromosomes I, II, IV, V, and X, again at different positions than in previous publications where QTLs for lifespan under DR were found in lines derived from N2 and CB4856 [70]. Population growth on the different bacteria, including the toxic Bt strains, did not match with the QTLs found for leaving behavior on those the same bacteria [36]. QTLs for body size similarly differed between studies. Snoek et al. [20] detected a QTL for body size (length) on chromosome IV whereas we found QTLs in other locations. For body size (volume), we found a QTL on chromosome X whereas Gutteling et al. [73] reported QTLs on chromosomes IV and X. These examples emphasize that our new multi-parent RIL panel shows high power to identify previously undetected QTLs for complex traits.

Our findings support the idea that a substantial part of the variation in a range of complex traits in *C. elegans* is determined by QTLs of large effect rather than only by multiple QTLs of small effect. This is corroborated by other studies mapping complex traits in *C. elegans*. McGrath et al. [51] identified two QTLs associated with digenic behavior in response to environmental O_2_ and CO_2_ levels. Gaertner et al. [37] mapped a set of few loci determining thermal preference and isothermal dispersion and found these loci to interact epistatically, explaining 50% of the total variation. Andersen et al. [46] reported the detection of a few QTLs for lifetime fecundity and adult body size explaining up to 23% of the variation. In an extensive complex trait mapping study, Andersen et al. [41] investigated the loci underlying fecundity and multiple body size traits. For fecundity, they found a single QTL on chromosome IV explaining 12% of the phenotypic variance. Comparing these results with the results obtained from the mpRILs shows that multiple and different variations inducing alleles, even with a relatively small effect, are present within the total set of genetic variation in *C. elegans.* Moreover, the allelic effects might be dependent on the genetic background or epistatic interactions [26].

The nematode is likely subject to boom-and-bust demographics in nature, which may or may not coincide with natural selection. These demographics can enhance genetic differentiation over time and space. As a consequence, RILs generated from genotypes, which have not yet been considered in previous panels, are likely genetically distinct, possibly helping to enhance our general ability to map QTLs. Moreover, if boom-and-bust demographics coincide with strong natural selection, then this could at least temporally lead to local adaptation. This may be expected if populations from different habitat types are considered, as it is the case here, where habitats are either more natural (wood habitat) or shaped by human activities (orchard), most likely leading to differences in the associated microbial communities, possible parasites, predators, and competitors, and also abiotic parameters. If such differences in natural selection exist, then the resulting mpRIL panel may help to identify the genetic basis of local adaptation.

The advantage of mpRIL populations to the classical 2 parental RIL populations is that mpRILs capture a larger part of natural genetic variants in more combinations and hence cover these variants better. This is supported by similar multi-parent RIL studies in *A. thaliana* populations derived from 19 different parental accessions [64], and many more MAGIC populations were developed for different species, including mice [65]. In our mpRIL population, the polymorphisms show more patterns of segregations due to SNP distribution patterns between the parental strains, making candidate/causal gene selections more efficient. As each recombination event can break up 7 SNP distribution patterns, we found 57 informative breakpoints per megabase pair, which can drastically reduce the number of candidate causal polymorphisms. These numbers suggest that our new panel may help to characterize the genetic architecture of complex traits at a higher resolution. Moreover, our developed 4-parent mpRIL population adds a new mapping tool for studying the complex trait architecture in the model species *C. elegans* and complements existing RIL panels using 2 parental strains and in case of the CeMEE panel 16 parents. Compared to the latter population, our mpRILs represent a relatively equal distribution of standing unperturbed local natural genetic variation as opposed to the genetic variation partially derived from laboratory selection experiments [26]. Taken together, our mpRIL population provides a straight forward and, in some cases, better performing alternative next to existing mapping panels.

## Conclusion

Overall, multi-parent RIL populations have a higher number of informative SNP markers than the classic two parental RIL sets in a variety of organisms. We show that in our mpRIL population, the number of QTLs is likely to be increased as well as the distinction of candidate causal SNPs and therefore resolution for genetic characterization of complex traits.

## Methods

### *C. elegans* strains, culturing, and crossing

*C. elegans* strains were cultured at 20 °C on OP50, unless specified otherwise for a specific screen or cross. For the construction of the multi-parental RIL, population lines were crossed as described in Fig. 1 and Additional file 1: Table S1 followed by 6 generations of single-worm descent inbreeding. Males were induced by heat stress (4–6 h at 30 °C). To allow the 4 parental genomes (JU1511, JU1941, JU1926, and JU1931) [25] to recombine, we set up a crossing scheme in which 2 pairs of wild isolates were crossed and both the obtained F1 populations were reciprocally inter-crossed (Fig. 1; Additional file 1: Table S1). To enable crossovers on chromosome X and to generate extra crossovers, the heterozygous F2 obtained from these initial crosses were further inter-crossed. To create homozygous genotypes, single worms were selected from the F2 as well as from the F2 inter-cross for 6 generations of single-worm inbreeding. From these 383 lines, a population of 200 different multi-parental recombinant inbred lines (mpRILs) was randomly picked for mRNA sequencing to obtain the genetic variation in the coding sequence.

### RNA sequencing

#### RNA isolation

For each mpRIL and parental strain, worms were grown on two 6-cm dishes at 16 °C on OP50 and bleached at the adult stage. The eggs were distributed over two 6-cm dishes and grown at 24 °C for 48 h after which the animals were rinsed of the plates and flash frozen in liquid nitrogen. We isolated RNA from these samples using the Maxwell® 16 AS2000 instrument with a Maxwell® 16 LEV simplyRNA Tissue Kit (both Promega Corporation, Madison, WI, USA). For isolation, the protocol was followed with a modified lysis step. In the lysis step, next to 200 μl homogenization buffer and 200 μl lysis buffer, 10 μl of a 20-mg/ml stock solution of proteinase K was added to each sample. Subsequently, the samples were incubated for 10 min at 65 °C and 1000 rpm in a Thermomixer (Eppendorf, Hamburg, Germany). After cooling on ice for 1 min, the standard protocol was followed.

#### Sequencing

We used standard Illumina protocols for the preparation and subsequent sequencing of RNA libraries. Libraries were sequenced on an Illumina HiSeq™ 2000 sequencing machine, using paired ends and 100 nucleotide read lengths. The raw data is available in the Sequence Read Archive (SRA; www.ncbi.nlm.nih.gov/bioproject/PRJNA495983/) with ID PRJNA495983.

#### SNP calling

The untrimmed paired-end reads were mapped against the N2 reference genome (WS220) [74, 75] using Tophat [76], allowing for four read mismatches and a read edit distance of 4. SNPs were called using SAMtools [77] mpileup with bcftools and vcfutils, using a minimum of five mapped reads per SNP. Further selection and quality filters were used in the construction of the genetic map to minimize the number and effect of false SNPs.

#### Construction of the genetic map

To construct a genetic map from the SNPs detected in the RNA-seq data, we adjusted the method used in Serin et al. [78]. For this *C. elegans* population, we selected the SNPs by several parameters. First, we selected those SNPs present in at least one of the parental lines in both RNA sequence replicates (two populations from separate plates were sequenced per parental line). Further selection was made based on (i) the presence in the mpRILs (min = 10, max = 180, quality > 199), (ii) correlation with neighboring SNPs of the same parental origin (> 0.8), and (iii) heterozygosity (< 40 mpRILs). These SNPs (Additional file 1: Table S2) were used directly in the SNP map of the population (Additional file 1: Table S3) or translated to the parental origin genetic map (Additional file 1: Table S4). For the parental origin map, stretches of ten SNPs were used to determine the parental origin and extended when inconclusive. In case of recombinations, breakpoints were put halfway between the determining SNPs.

### Phenotyping

#### Population growth

Orsay/Santeuil mpRIL population growth was measured as the total offspring of three L4 hermaphrodites after 5 days at 20 °C in liquid peptone free medium (PFM). 24-well plates were inoculated with 1 ml liquid PFM per well and food bacteria added to a final OD_600_ of 5. The six different bacterial treatments were (i) *Escherichia coli OP50*, (ii) *Erwinia rhapontici* (isolated from Orsay, France), (iii) *Sphingobacterium* sp. (isolated from Orsay, France), (iv) a non-pathogenic *Bacillus thuringiensis* strain DSM-350E, and a pathogenic *Bacillus thuringiensis* strain NRRL B-18247 in the two concentrations of (v) 1:300 and (vi) 1:600. After 5 days, worms were fixed in 4% formaldehyde and stored at 8 °C until counting [25].

#### Lifespan assays

Worm lifespan assays were performed at 20 °C, with populations initiated from synchronized larvae isolated by incubating the eggs from sodium hypochlorite-treated gravid adults on plates without a food source [42]. After 24 h, the plates were seeded with *Escherichia coli* OP50 as a food source and the worms were allowed to grow, en masse, for 48 h to the L4/young adult’s stage. After 48 h, ad libitum (normal lifespan) worms were moved to fresh seeded standard NGM plates (5 worms per plate and 8 plates per treatment). The lifespan under DR worms was moved to seeded PFM plates (5 worms per plate and 8 plates per treatment). The method of total withdrawal of peptone from the agarose plates is a relatively mild form of DR, as described by Stastna et al. [70]. To test the lifespan, worms were observed daily, with nematodes transferred to new plates every day until reproduction had ceased as assays were performed without the use of FUdR. After the reproductive period, the DR worms were moved to fresh plates every other day to prevent food deprivation. Worms were considered to have died if they were not moving and failed to respond to touch with a worm pick. Any worms that died due to maternal hatching (bagging) were censored out of the analysis of lifespan. Each mpRIL within an experimental block was tested at the same time under both conditions, with a total of 40 worms per treatment per mpRIL; plates were then randomized and blind coded. The movement of the L4/young adult worms to fresh plates was counted as day 1 for all the lifespan measurements. In total, the mpRILs were assayed in 6 blocks with 35–48 formally randomly selected mpRILs in each block, with some mpRILs present in multiple blocks. RILs were not included in the analysis if the lifespan of less than 3 worms was observed per treatment. In addition to the mpRILs, the 4 parental lines and N2 were also tested in all lifespan assays.

#### Heat shock resistance

Worms were cultured at 15 °C prior to the heat shock assays. Worms were synchronized as for the lifespan assay and allowed to grow en masse to L4/young adult stage [42, 79, 80]. At this stage, worms were transferred to fresh plates, ten worms per plate with five replicates for each of the mpRILs and each of the parental strains. The plates were then randomized and blind coded. Worms were then placed at 35 °C for 10 h. After the heat shock, worms were allowed to rest at 15 °C for 48 h before scoring for survival, when worms that did not respond to a gentle prod with the worm pick were scored as dead. Worms that crawled off the plates or died of bagging were censored from the experiment. The data were then converted into a proportion of survival.

#### Oxidative stress resistance

Worms were maintained at 20 °C, synchronized as described above, and grown en masse to the L4/young adult’s stage. After 48 h, the worms were washed off the plates with M9 buffer and 10–30 individuals were transferred to 96-well plates in a total volume of 48 μl, with 3 replicates for all the mpRILs and N2. The plates were then transferred to a WMicrotracker-One™ (PhylumTech), and activity over 30 min was determined at 20 °C. After this step, 2 μl of 0.4% H_2_O_2_ solution was added to all wells, giving a final volume of 50 μl, except for the control, which had 2 μl of M9 buffer added to make up the final volume. Worms were then incubated for 24 h at 20 °C. After 24 h, the locomotive activity of the worms was measured again. WMicrotracker-One™ records the movement as photo beam interruptions within wells of 96-well plates. The data were then processed as follows: (activity of the wells before − activity after 24 h)/before = activity score. An activity score of − 1 represents no movement and hence that all worms were dead at the end of the treatment, and an activity score of 0 indicates an activity level after 24 h that is the same as before. This score can also generate values above 0, which indicates that worms were more active after the hydrogen peroxide treatment.

#### Developmental time and occurrence of males

Starvation-synchronized L1 juveniles of the mpRILs and parental strains were grown on *E. coli* OP50 at 24 °C and after 48 h inspected at 1-h time intervals. Developmental time was defined as the period between synchronized hatching and time until the first eggs. Time until first egg scoring was adjusted from [25, 81] by placing 20–40 worms on NGM, done *in duplo*, and scoring every hour starting at 48 h until 54 h for eggs. This was done on *E. coli* OP50 and *Erwinia rhapontici* bacteria previously used to measure the time until the first egg in multiple wild isolates [25]. We scored the time of the first egg visible on plate and the time when multiple groups of ~ 10 eggs were visible. Averages of these time points per mpRIL were used in QTL mapping. Moreover, the occurrence of males on the plates was recorded after population growth and used for QTL mapping.

#### Size and volume

Analysis of the length and width of young gravid adults of the mpRILs was performed with a particle analyzer (RapidVue; Beckman Coulter Inc., Miami, FL, USA) [25]. Phenotypic size and volume data for the parental strains could not be recorded together with the mpRILs. The size and volume of the parental strains were measured in Volkers et al. 2013, yet due to the between-batch variation, these measurements are incomparable to the mpRILs measured in this study. The parental measurements were not needed for the QTL mapping.

### QTL mapping

We started with single-marker mapping for each trait to find the SNP with the most significant QTL (Additional file 5: Figure S4). This SNP was used as the starting point in the forward mapping approach. Forward mapping was done by selecting co-factors one by one, starting with the most significant and remap with that co-factor, and selecting the next most significant SNP until no more SNP was present with a *p* < 0.001 or a maximum of ten co-factors was reached. Then, QTLs were remapped with the selected co-factors and an exclusion window of 2 Mbp. Co-factors within this window were excluded from the mapping model when QTLs were mapped in the window (Additional file 4: Figure S3). Obtained QTLs were determined significant when −log10(*p*) > 3 and borders were determined at the point where the QTL profile drops 2 −log10(*p*) scores below the peak. (Permutations showed maximum QTLs ranging from −log10(*p*) of 2.1 to 3.9, with the exception of the traits describing the occurrence of males on the plate for which in a number of permutations, a −log10(*p*) was found > 4.5). All QTL profiles can be obtained and interactively explored in EleQTL (www.bioinformatics.nl/EleQTL). The heritability for each trait was calculated by dividing the variation between the mpRILs by the total variation.

## Additional files

Additional file 1: **Table S1.** Detailed crossing scheme used to make the mpRILs. The crosses from which each individual mpRIL was made can be found here. **Table S2.** SNP info. SNP position and SNP distribution pattern (SDP). **Table S3.** SNP genetic map. SNP identity per mpRIL. **Table S4.** Parental background genetic map. Genome-wide parental background for each individual mpRIL. **Table S5.** Average phenotypes per mpRIL used for QTL mapping. Including WN and sequence identifiers. **Table S6.** Trait descriptive. Number of mpRILs for which the phenotype was measured, minimum trait value, maximum trait value, mean trait value, median trait value, trait value of parental line JU1511, trait value of parental line JU1926, trait value of parental line JU1931, trait value of parental line JU1941, heritability, heritability type, number of QTLs found, and explained variation by QTLs. **Table S7.** Correlation between traits. Pearson correlation of the mean traits values in the mpRIL population. **Table S8.** Identified QTLs. (XLSX 11929 kb)

Additional file 2: **Figure S1.** Distribution of parental alleles in the multi-parental recombinant inbred lines. Colors indicate the percentage of parental occurrence per genomic position (*x*-axis) per cross (*y*-axis) as estimated from the parental SNP distribution patterns (SDP). Chromosomes are in separate panels on the *x*-axis. mpRILs are grouped according to their cross history. Group Z is the parental lines. (PDF 536 kb)

Additional file 3: **Figure S2.** SNP distribution pattern (SDP) per mpRIL. For each of the seven SDPs, the genotype for each mpRIL is shown. SDPs are shown on top, chromosomes on the right. The genotype of the mpRIL is green when it has the SNP corresponding to the SDP and red when it has the opposite variant. SDP 12 (JU1511/JU1926 vs JU1931/JU1941), 13 (JU1511/JU1931 vs JU1926/JU1941), 14 (JU1511/JU1941 vs JU1926/JU1931), JU1511 (JU1511 vs rest), JU1926 (JU1926 vs rest), JU1931 (JU1931 vs rest), and JU1941 (JU1941 vs rest). Notice that each recombination event can break up multiple SDP. (TIFF 11718 kb)

Additional file 4: **Figure S3.** Forward mapping QTL profiles for each trait. Trait names are shown on the right. Chromosome number is shown on top. Genomic position in megabase pair is shown on the *x*-axis. For each SNP, the significance in −log10(*p*) is multiplied by the sign of the effect on the *y*-axis. Colors indicate SPD of the SNP and triangle the co-factors used in the final model of the forward mapping approach. (PDF 883 kb)

Additional file 5: **Figure S4.** Single marker QTL profiles for each trait. Trait names are shown as title. Chromosome number is shown on top. Genomic position in megabase pair is shown on the *x*-axis. For each SNP, the significance in −log10(*p*) is multiplied by the sign of the effect on the *y*-axis. Colors indicate SPD of the SNP. (PDF 891 kb)
