# Supplementary figures and images for: A multi-parent recombinant inbred line population of C. elegans allows identification of novel QTLs for complex life history traits
Source: BMC Biol. 2019 Mar 12;17:24. doi: 10.1186/s12915-019-0642-8 (PMC6417139; doi:10.1186/s12915-019-0642-8)

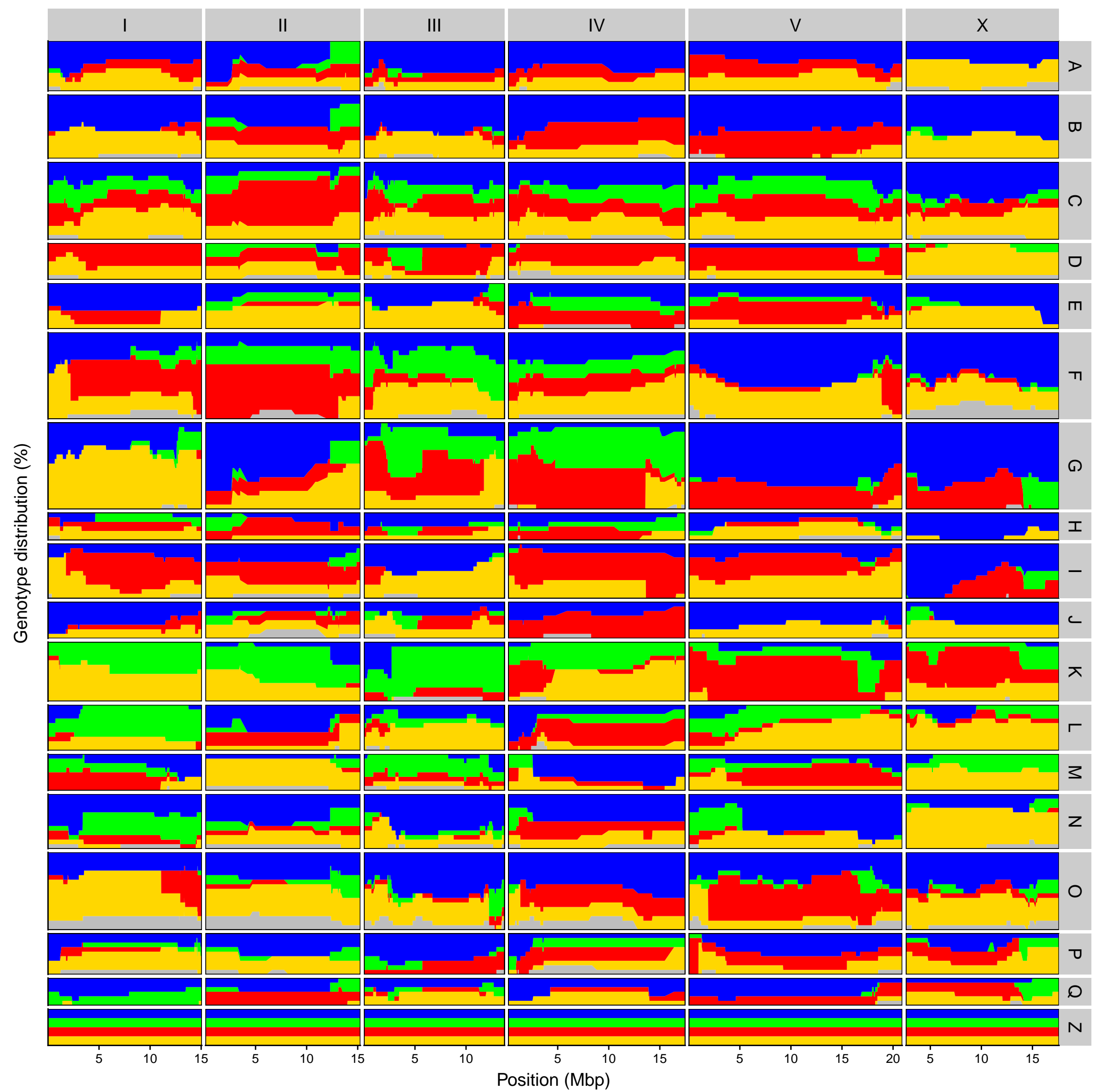

Supplement: Supplementary file 2 — Figure S1. Distribution of parental alleles in the multi-parental recombinant inbred lines. Colors indicate the percentage of parental occurrence per genomic position (x-axis) per cross (y-axis) as estimated from the parental SNP distribution patterns (SDP). Chromosomes are in separate panels on the x-axis. mpRILs are grouped according to their cross history. Group Z is the parental lines. (PDF 536 kb) [file 12915_2019_642_MOESM2_ESM.pdf]

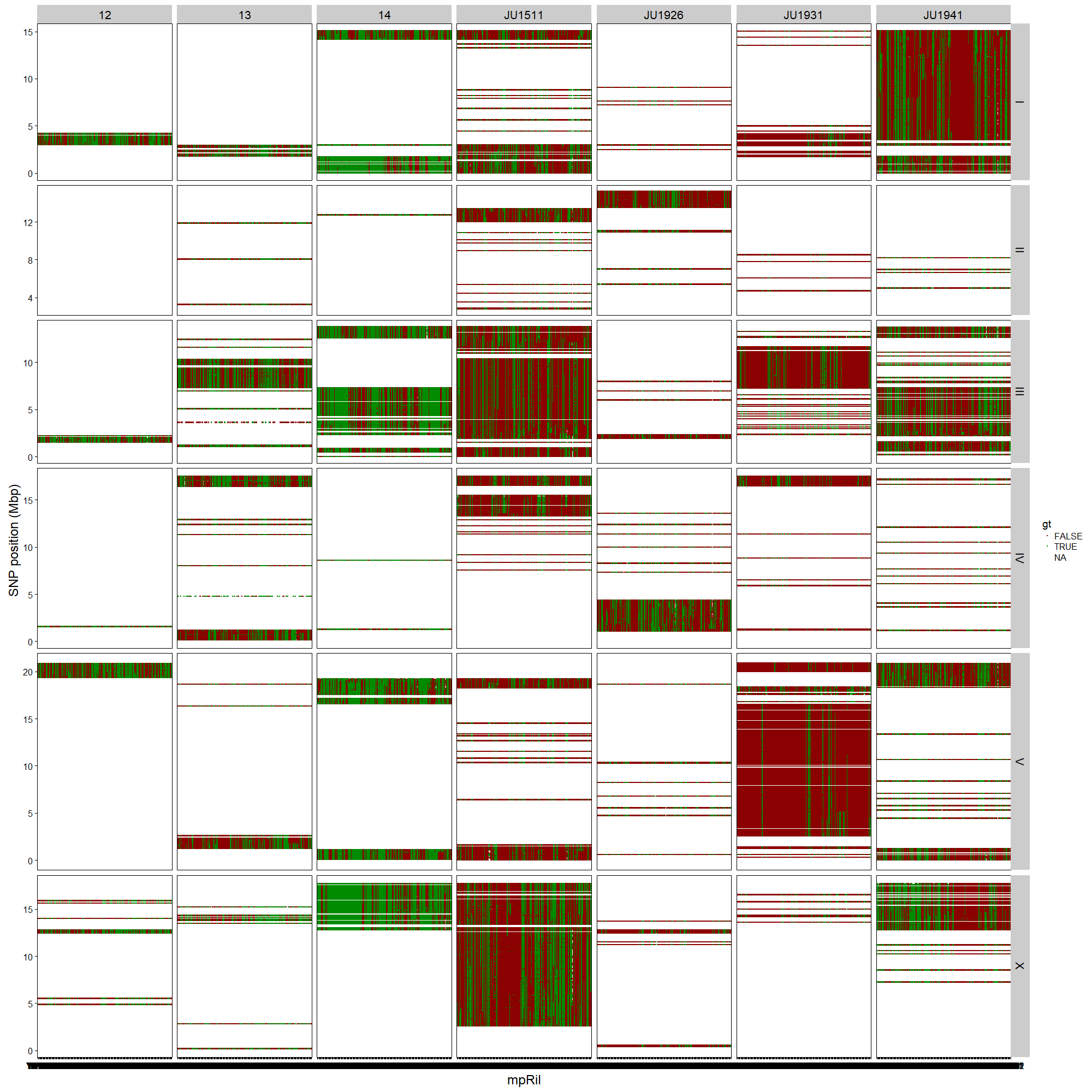

Supplement: Supplementary file 3 — Figure S2. SNP distribution pattern (SDP) per mpRIL. For each of the seven SDPs, the genotype for each mpRIL is shown. SDPs are shown on top, chromosomes on the right. The genotype of the mpRIL is green when it has the SNP corresponding to the SDP and red when it has the opposite variant. SDP 12 (JU1511/JU1926 vs JU1931/JU1941), 13 (JU1511/JU1931 vs JU1926/JU1941), 14 (JU1511/JU1941 vs JU1926/JU1931), JU1511 (JU1511 vs rest), JU1926 (JU1926 vs rest), JU1931 (JU1931 vs rest), and JU1941 (JU1941 vs rest). Notice that each recombination event can break up multiple SDP. (TIFF 11718 kb) [file 12915_2019_642_MOESM3_ESM.tiff]

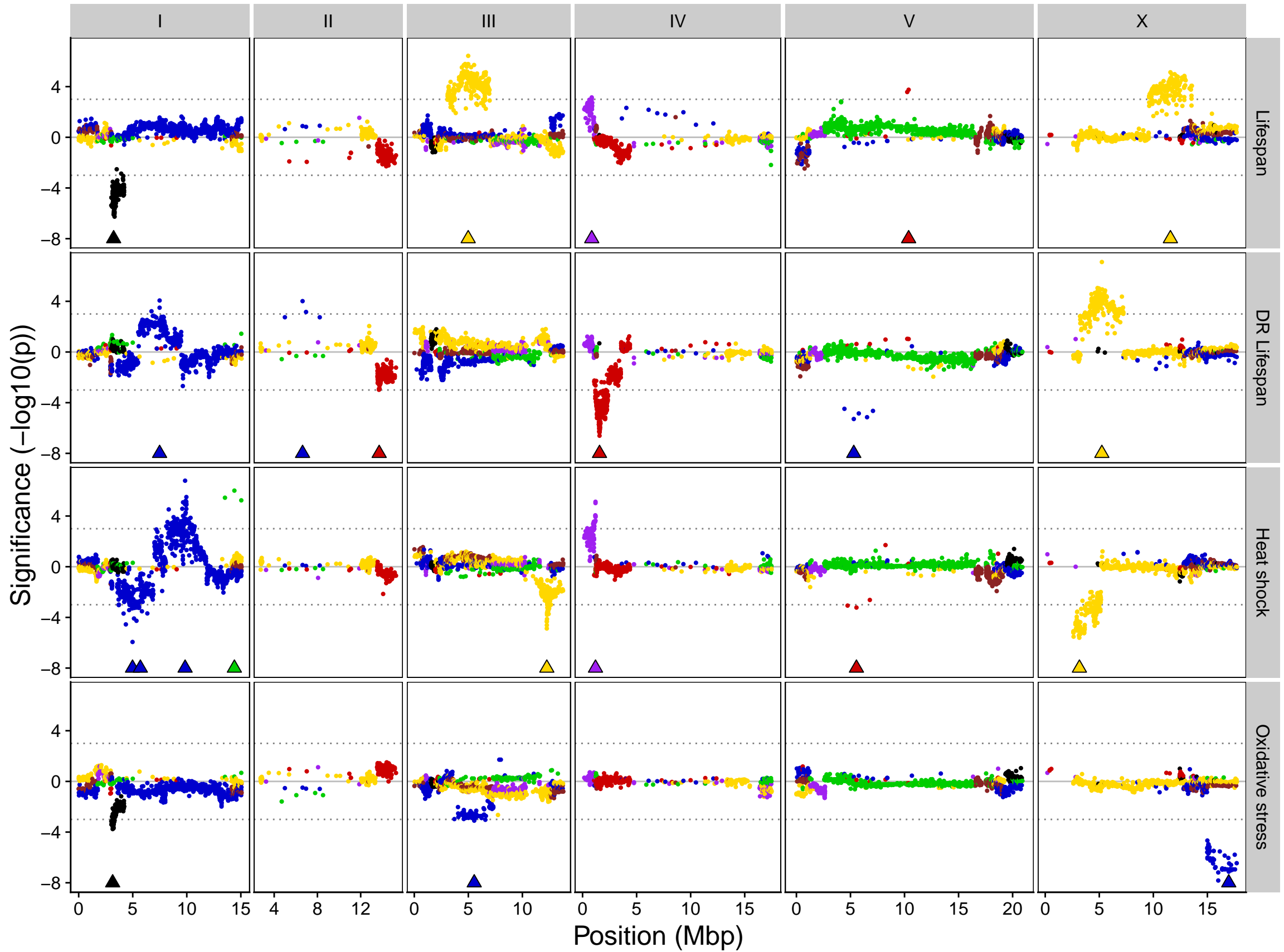

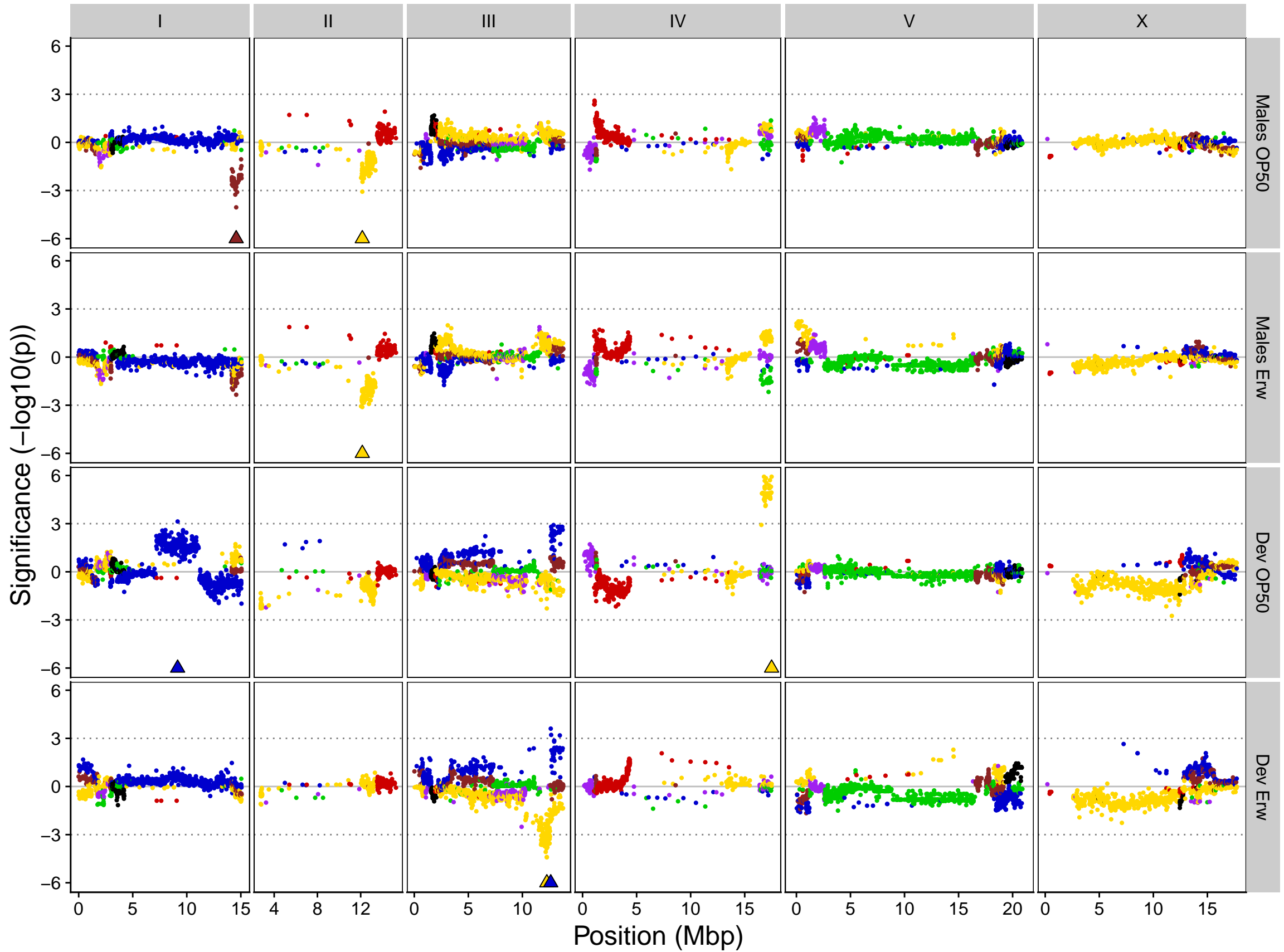

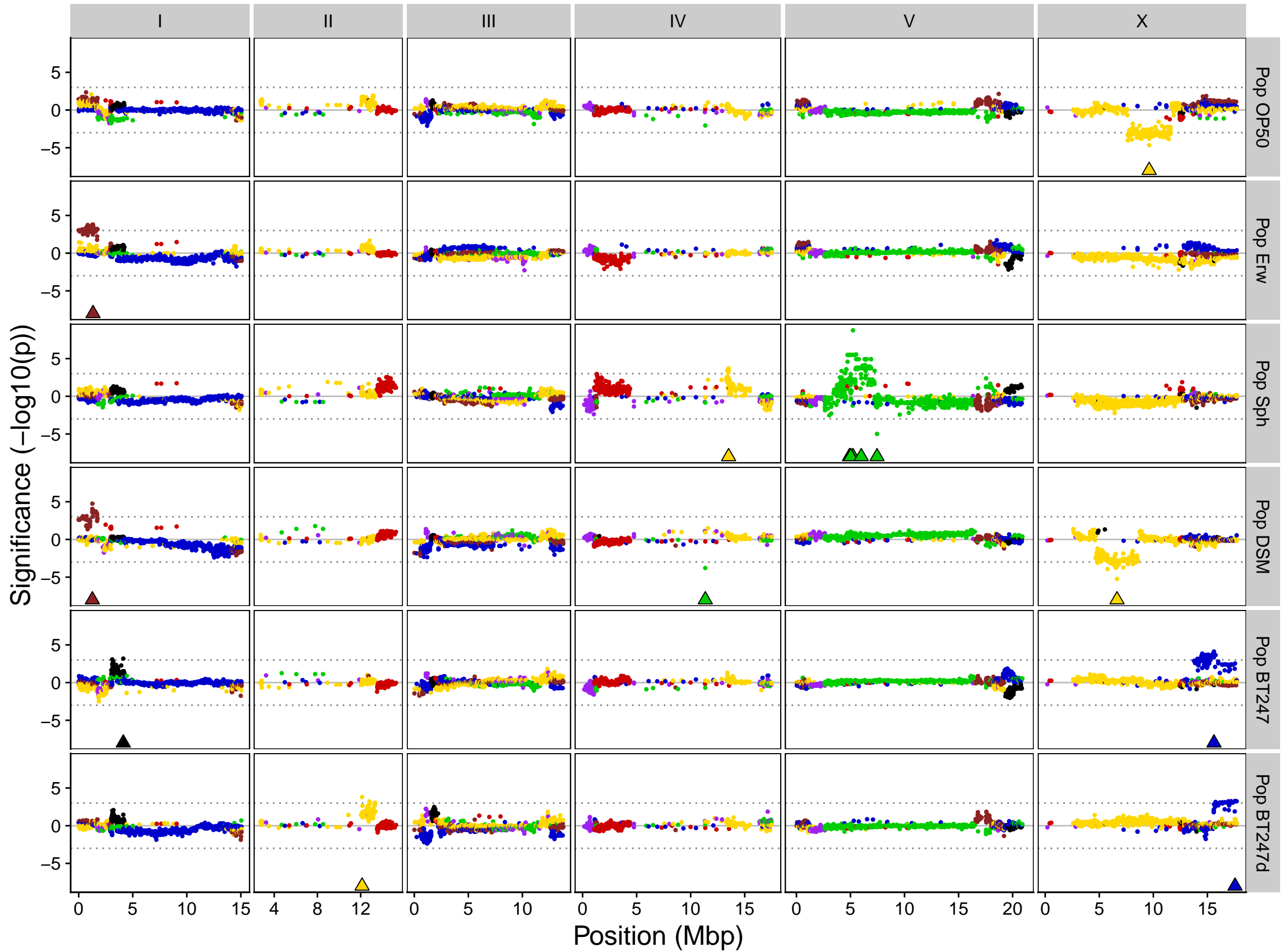

SDP

|      |          |          |          |
|------|----------|----------|----------|
| ▲ 12 | ▲ 14     | ▲ JU1926 | ▲ JU1941 |
| ▲ 13 | ▲ JU1511 | ▲ JU1931 |          |

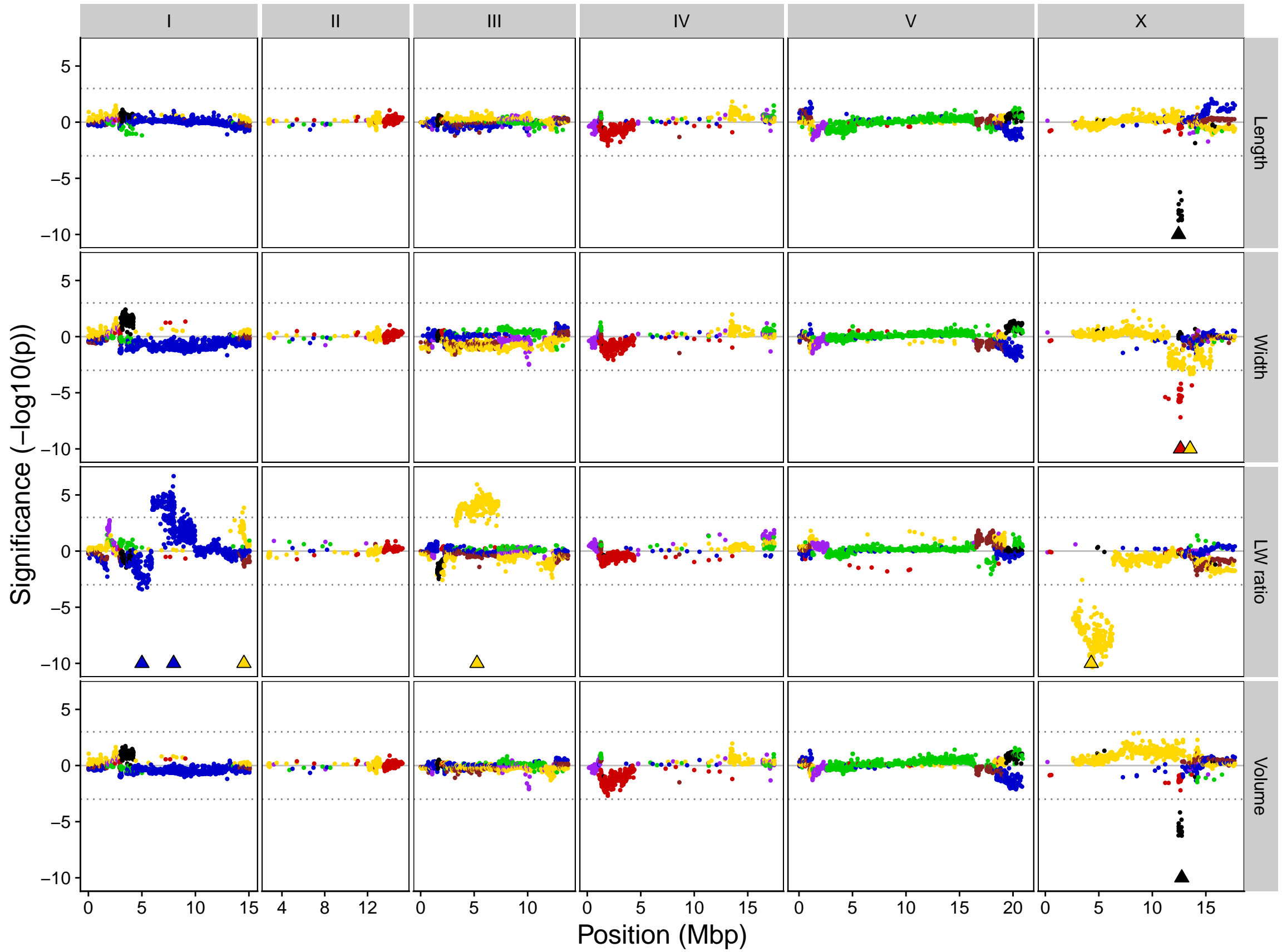

Supplement: Supplementary file 4 — Figure S3. Forward mapping QTL profiles for each trait. Trait names are shown on the right. Chromosome number is shown on top. Genomic position in megabase pair is shown on the x-axis. For each SNP, the significance in −log10(p) is multiplied by the sign of the effect on the y-axis. Colors indicate SPD of the SNP and triangle the co-factors used in the final model of the forward mapping approach. (PDF 883 kb) [file 12915_2019_642_MOESM4_ESM.pdf]
