# Supplementary material for: A multi-parent recombinant inbred line population of C. elegans allows identification of novel QTLs for complex life history traits
Source: BMC Biol. 2019 Mar 12;17:24. doi: 10.1186/s12915-019-0642-8 (PMC6417139; doi:10.1186/s12915-019-0642-8)
Supplement: Supplementary file 5 — Figure S4. Single marker QTL profiles for each trait. Trait names are shown as title. Chromosome number is shown on top. Genomic position in megabase pair is shown on the x-axis. For each SNP, the significance in −log10(p) is multiplied by the sign of the effect on the y-axis. Colors indicate SPD of the SNP. (PDF 891 kb) [file 12915_2019_642_MOESM5_ESM.pdf]

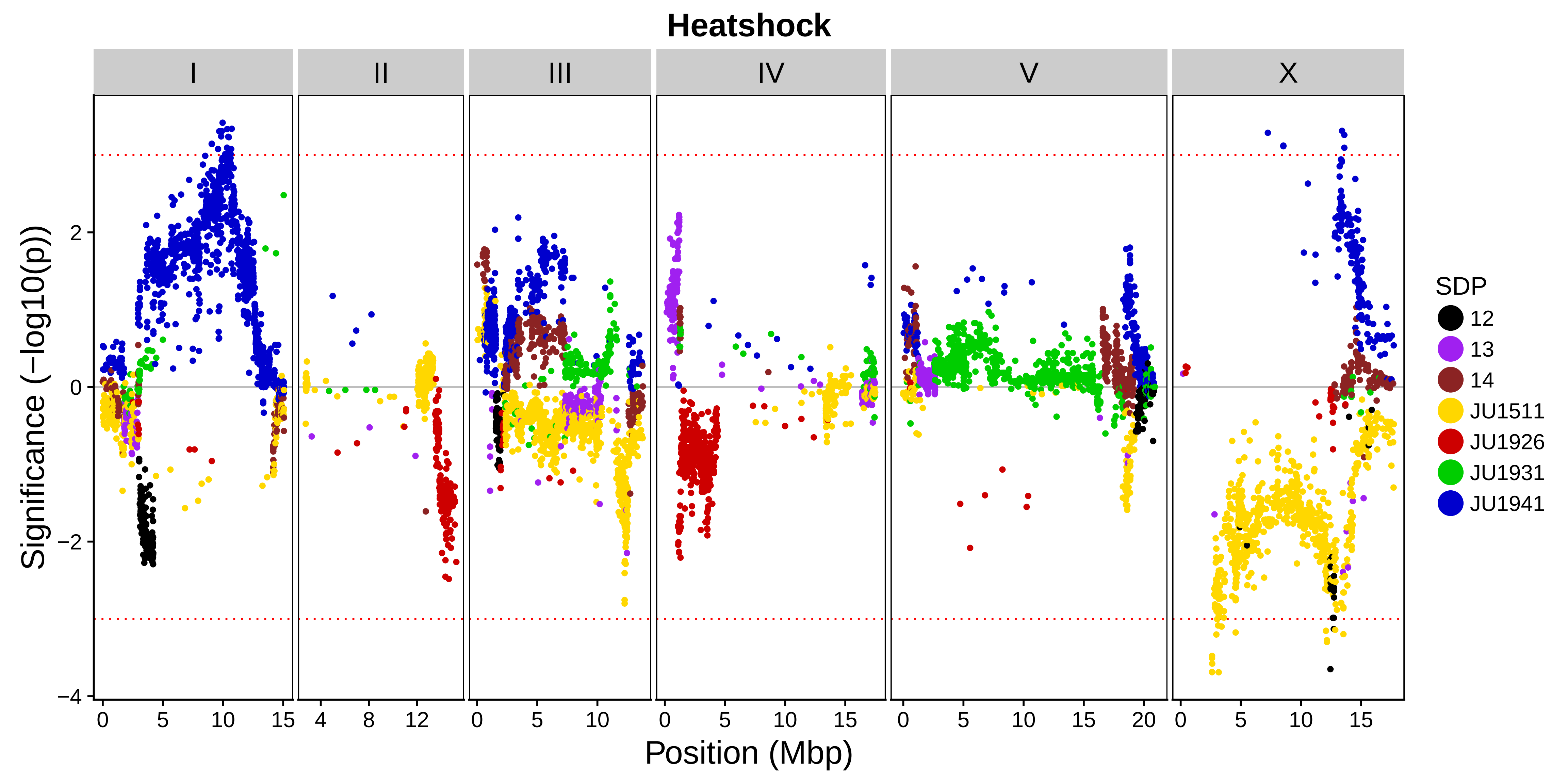

Heatshock
I
II
III
IV
V
X
Significance (−log10(p))
Position (Mbp)
SDP
12
13
14
JU1511
JU1926
JU1931
JU1941

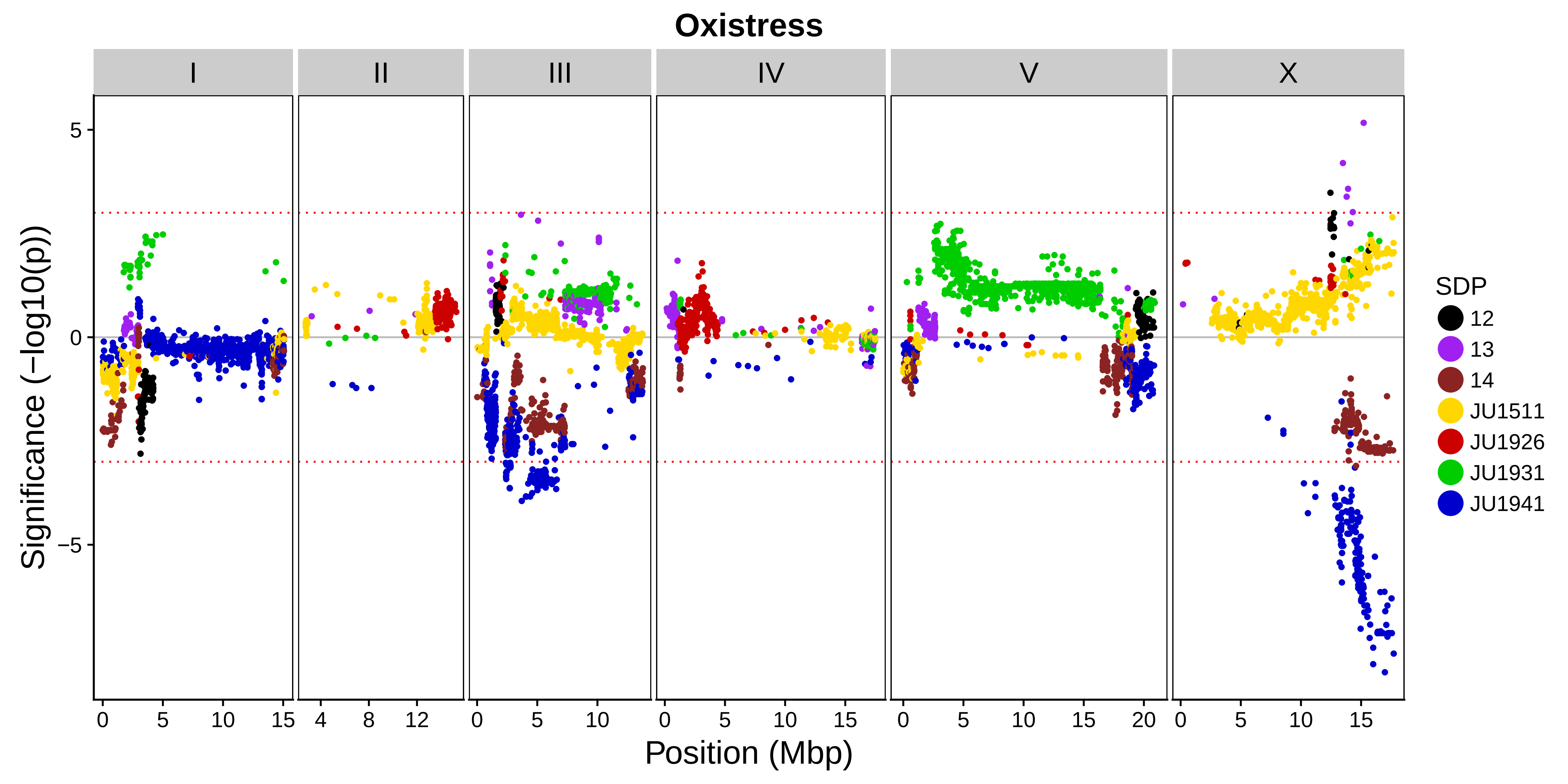

Oxistress
I
II
III
IV
V
X
Significance (−log10(p))
Position (Mbp)
SDP
12
13
14
JU1511
JU1926
JU1931
JU1941

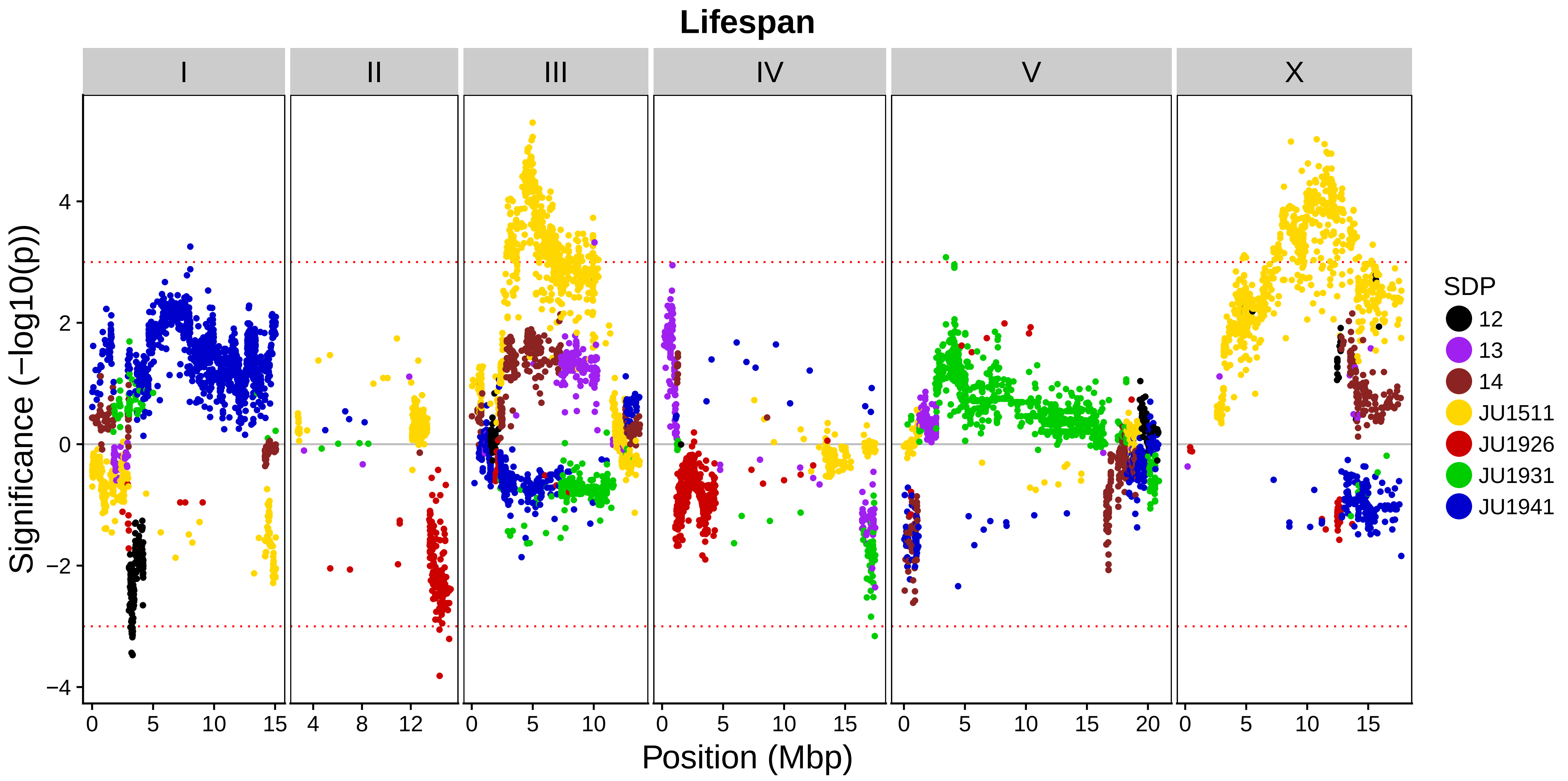

Lifespan
I
II
III
IV
V
X
Significance (−log10(p))
4
2
0
−2
−4
0
5
10
15
4
8
12
0
5
10
0
5
10
15
0
5
10
15
20
0
5
10
15
Position (Mbp)
SDP
12
13
14
JU1511
JU1926
JU1931
JU1941

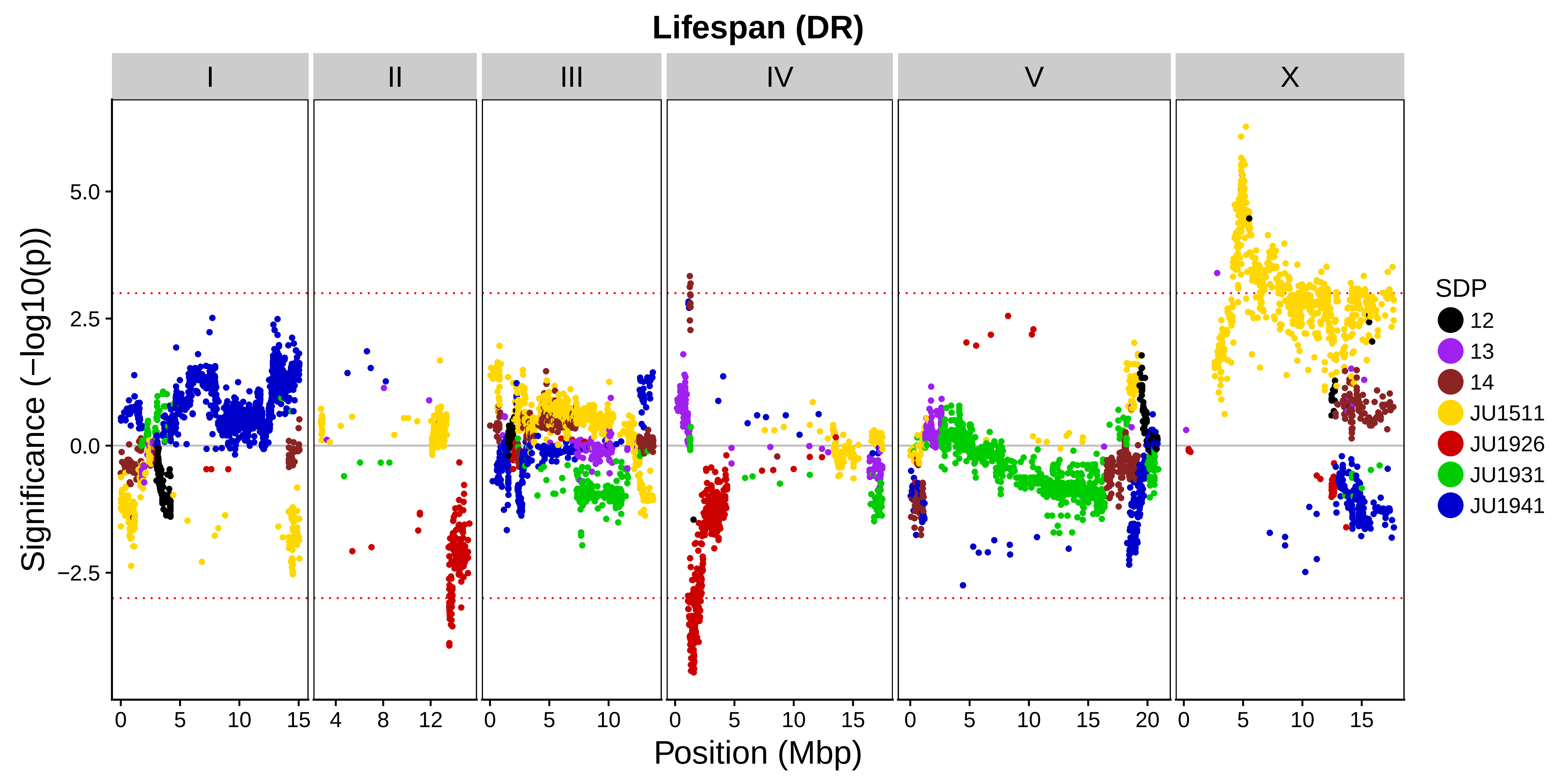

Lifespan (DR)
I
II
III
IV
V
X
Significance (−log10(p))
5.0
2.5
0.0
−2.5
0
5
10
15
4
8
12
0
5
10
0
5
10
15
0
5
10
15
20
0
5
10
15
Position (Mbp)
SDP
12
13
14
JU1511
JU1926
JU1931
JU1941

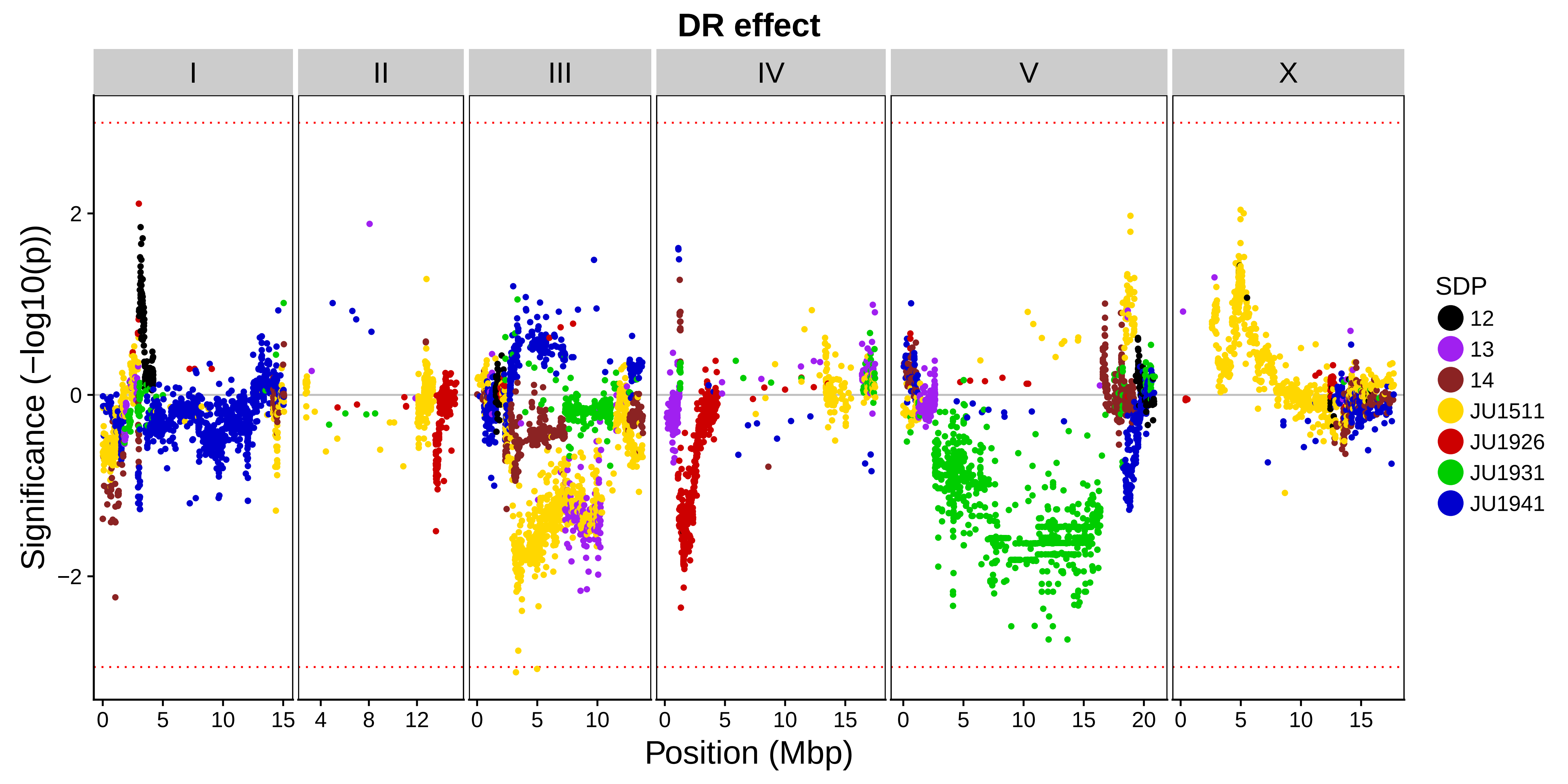

DR effect
I
II
III
IV
V
X
Significance (−log10(p))
2
0
−2
0
5
10
15
4
8
12
0
5
10
0
5
10
15
0
5
10
15
20
0
5
10
15
Position (Mbp)
SDP
12
13
14
JU1511
JU1926
JU1931
JU1941

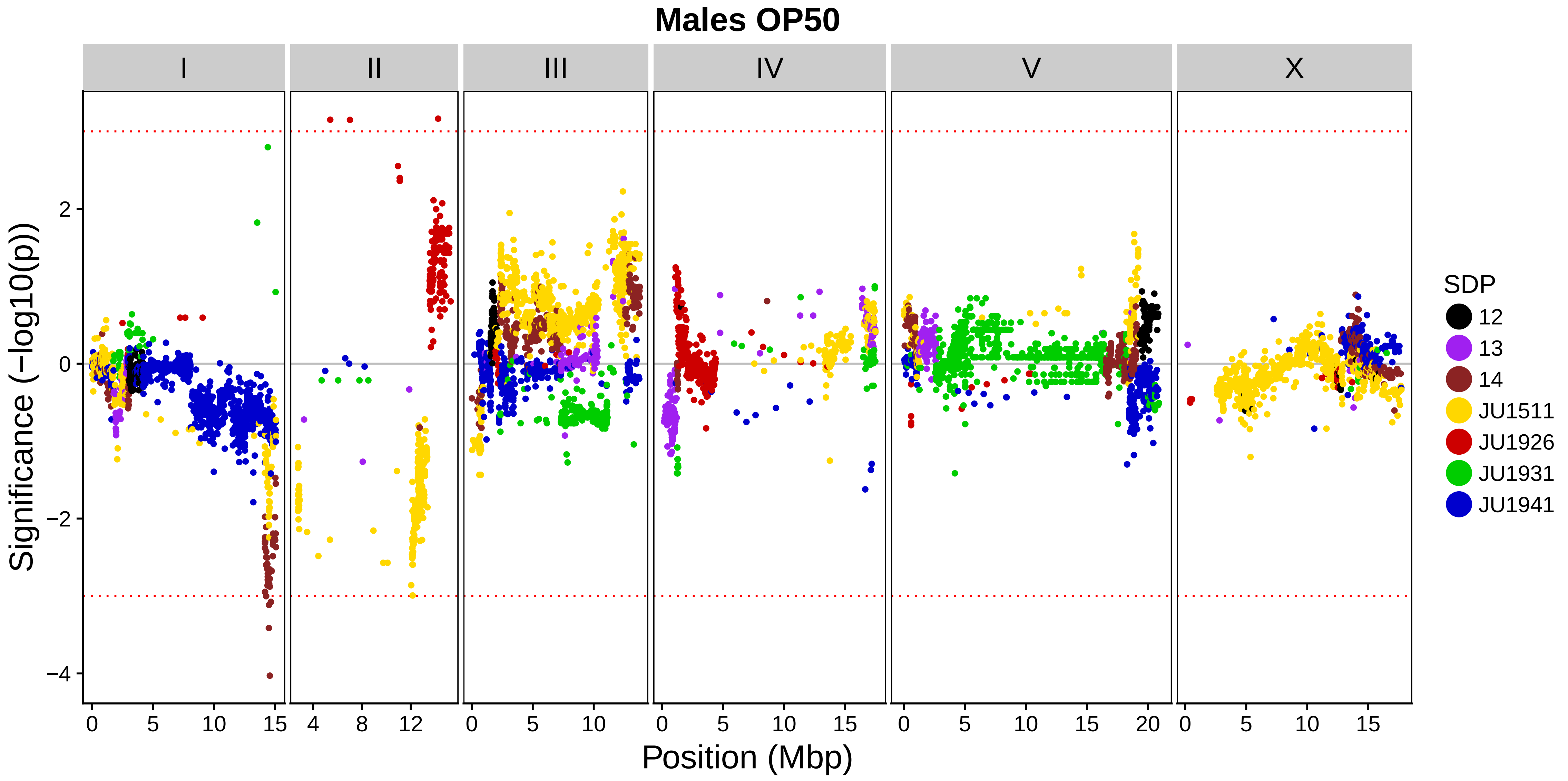
Males OP50
I
II
III
IV
V
X
Significance (−log10(p))
2
0
−2
−4
0
5
10
15
4
8
12
0
5
10
0
5
10
15
0
5
10
15
20
0
5
10
15
Position (Mbp)
SDP
12
13
14
JU1511
JU1926
JU1931
JU1941

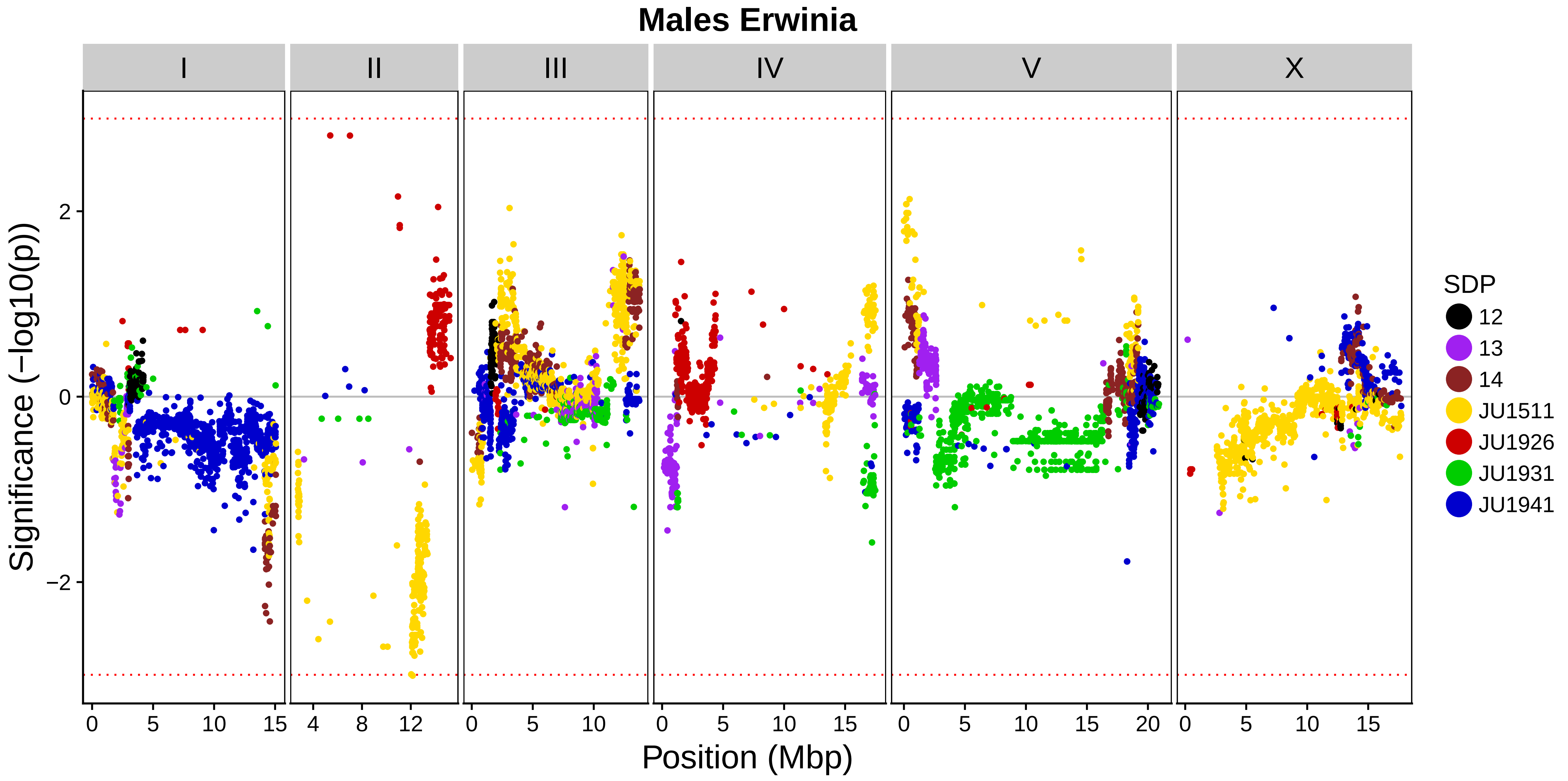

Males Erwinia
I
II
III
IV
V
X
Significance (−log10(p))
2
0
−2
Position (Mbp)
0
5
10
15
4
8
12
0
5
10
0
5
10
15
0
5
10
15
20
0
5
10
15
SDP
12
13
14
JU1511
JU1926
JU1931
JU1941

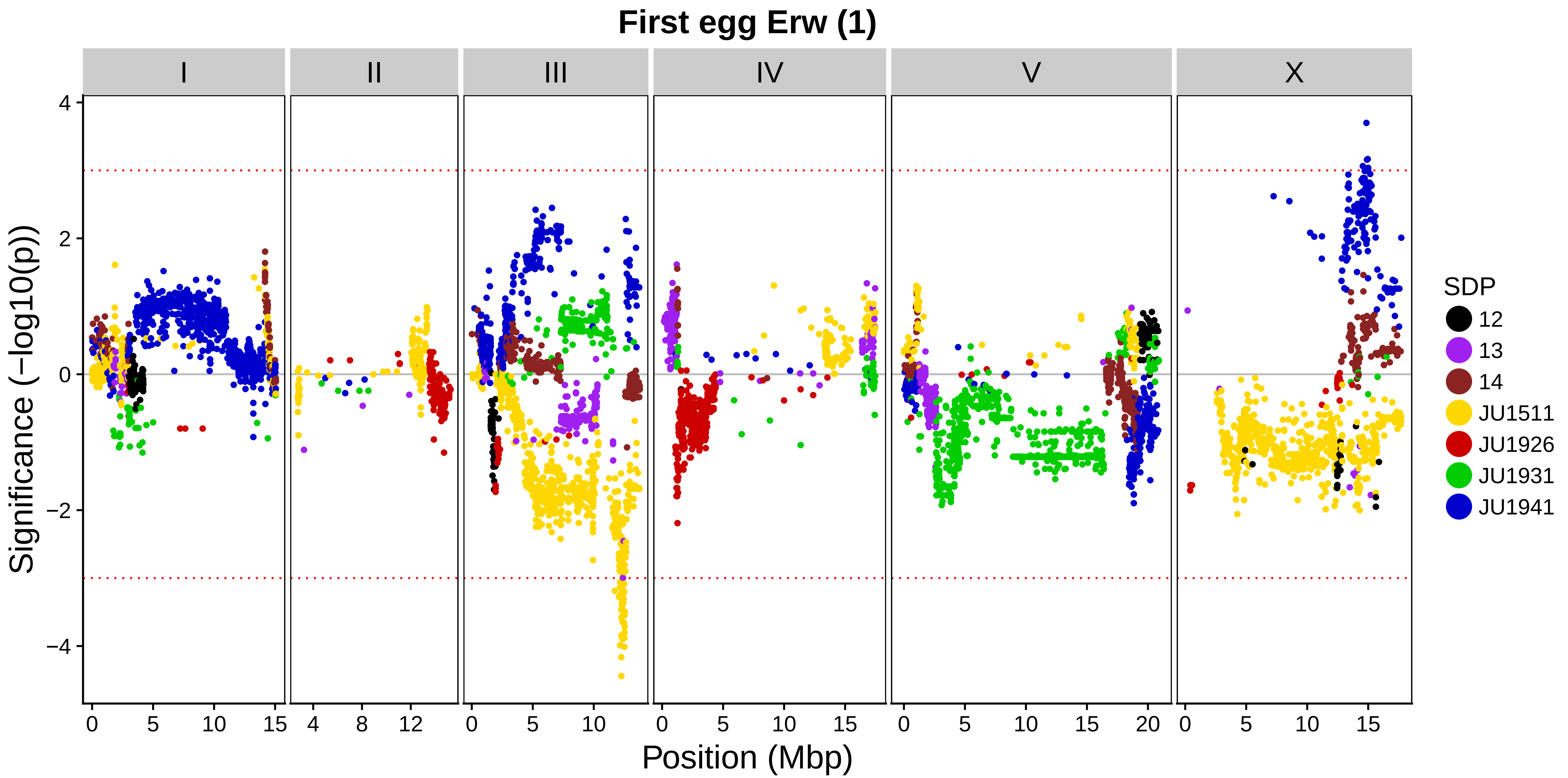

First egg Erw (1)
I
II
III
IV
V
X
Significance (−log10(p))
Position (Mbp)
SDP
12
13
14
JU1511
JU1926
JU1931
JU1941

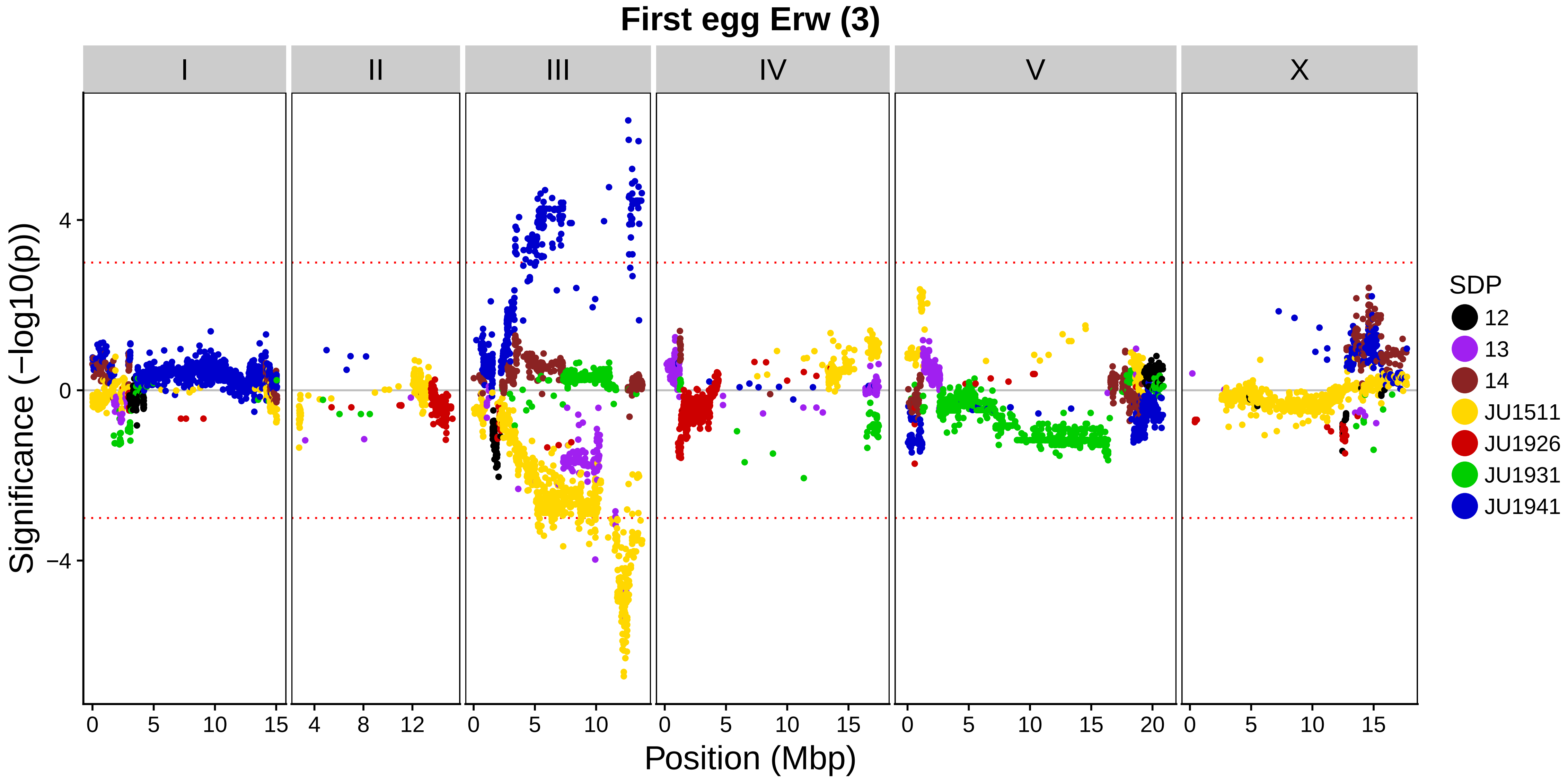
First egg Erw (3)
I
II
III
IV
V
X
Significance (−log10(p))
4
0
−4
0
5
10
15
4
8
12
0
5
10
0
5
10
15
0
5
10
15
20
0
5
10
15
Position (Mbp)
SDP
12
13
14
JU1511
JU1926
JU1931
JU1941

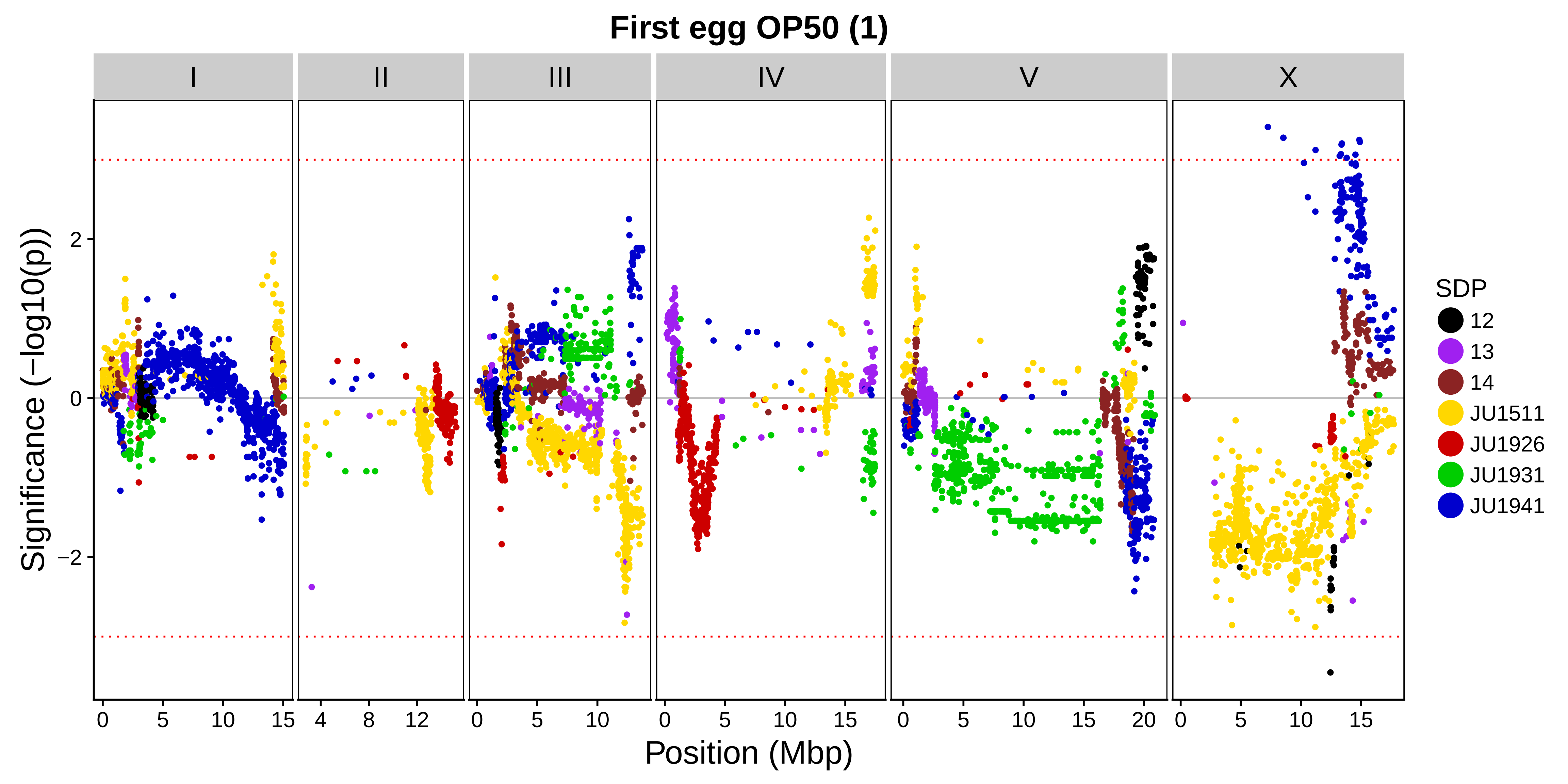

First egg OP50 (1)
I
II
III
IV
V
X
Significance (−log10(p))
Position (Mbp)
SDP
12
13
14
JU1511
JU1926
JU1931
JU1941

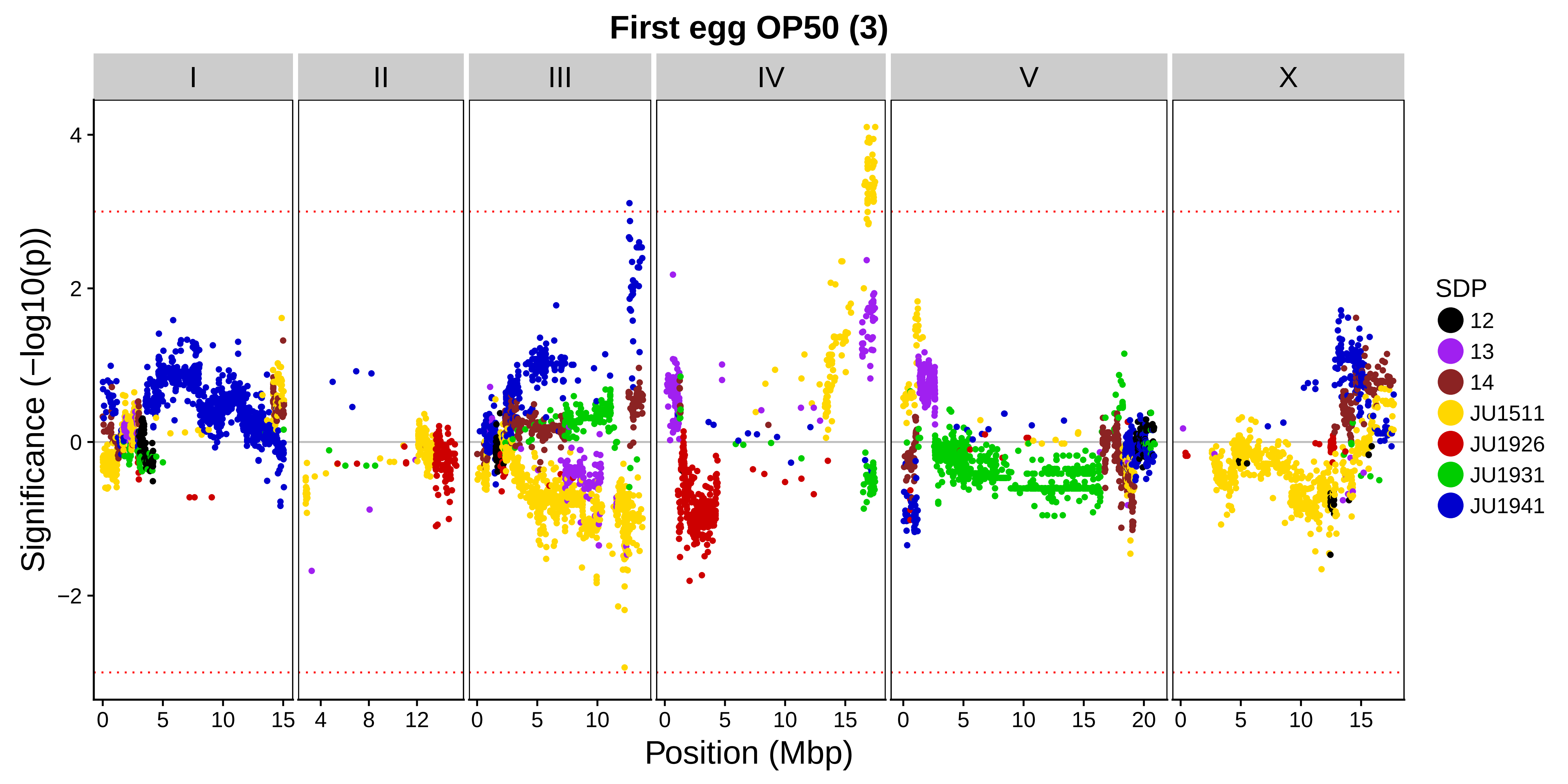

First egg OP50 (3)
I
II
III
IV
V
X
Significance (−log10(p))
4
2
0
−2
0
5
10
15
4
8
12
0
5
10
0
5
10
15
0
5
10
15
20
0
5
10
15
Position (Mbp)
SDP
12
13
14
JU1511
JU1926
JU1931
JU1941

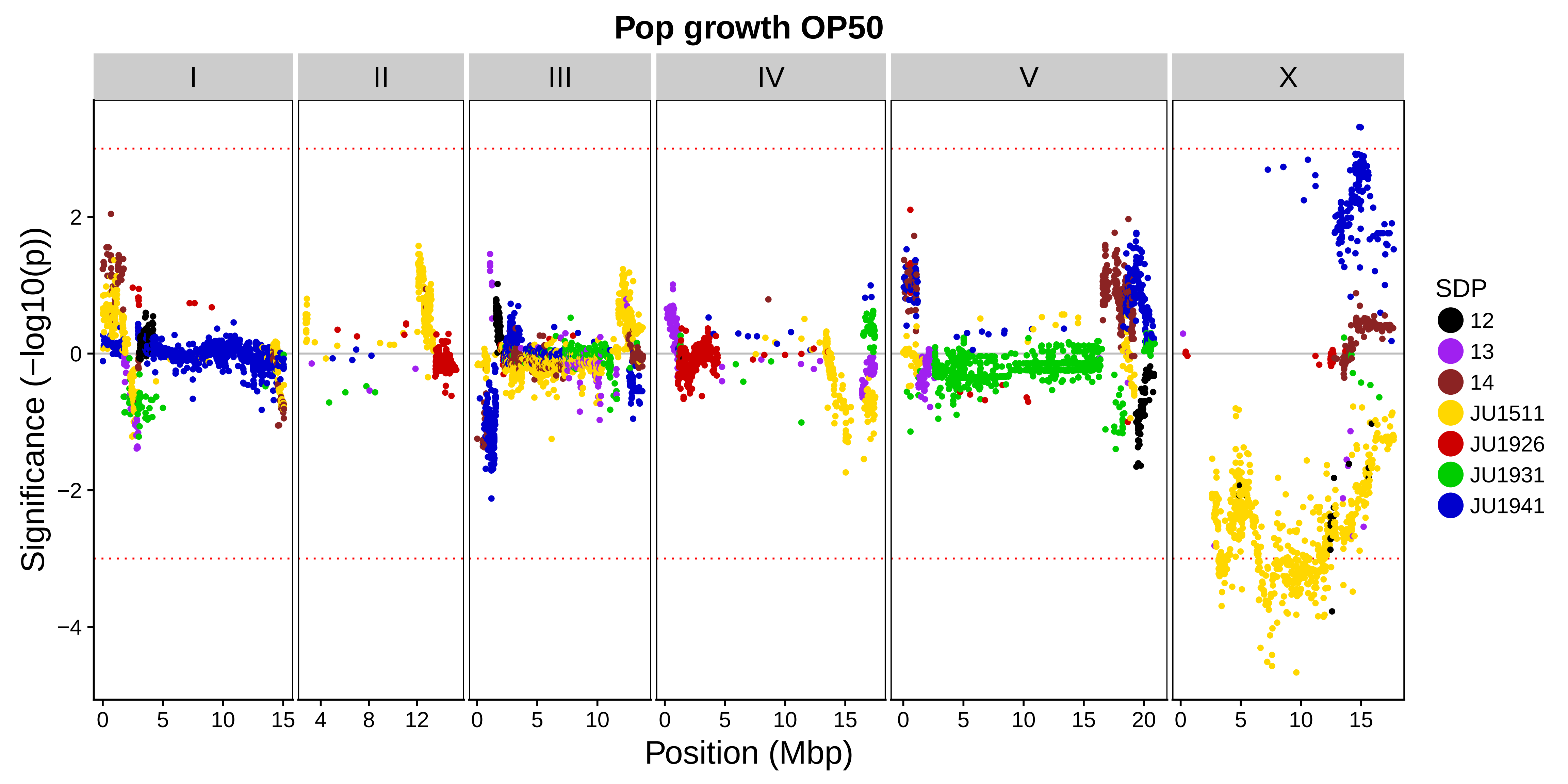

Pop growth OP50
I
II
III
IV
V
X
Significance (−log10(p))
Position (Mbp)
SDP
12
13
14
JU1511
JU1926
JU1931
JU1941

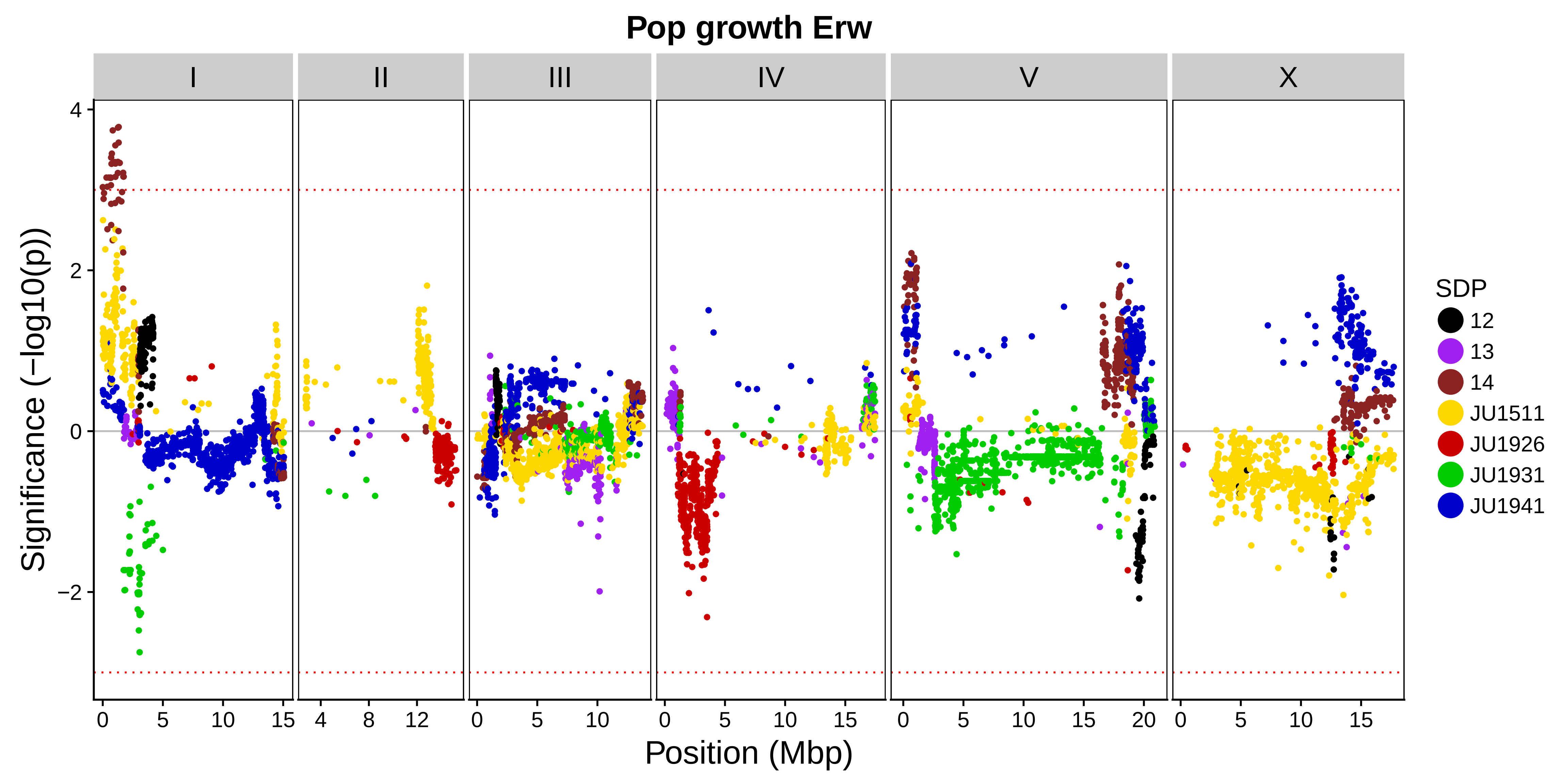

Pop growth Erw
I
II
III
IV
V
X
Significance (−log10(p))
4
2
0
−2
Position (Mbp)
0
5
10
15
4
8
12
20
SDP
12
13
14
JU1511
JU1926
JU1931
JU1941

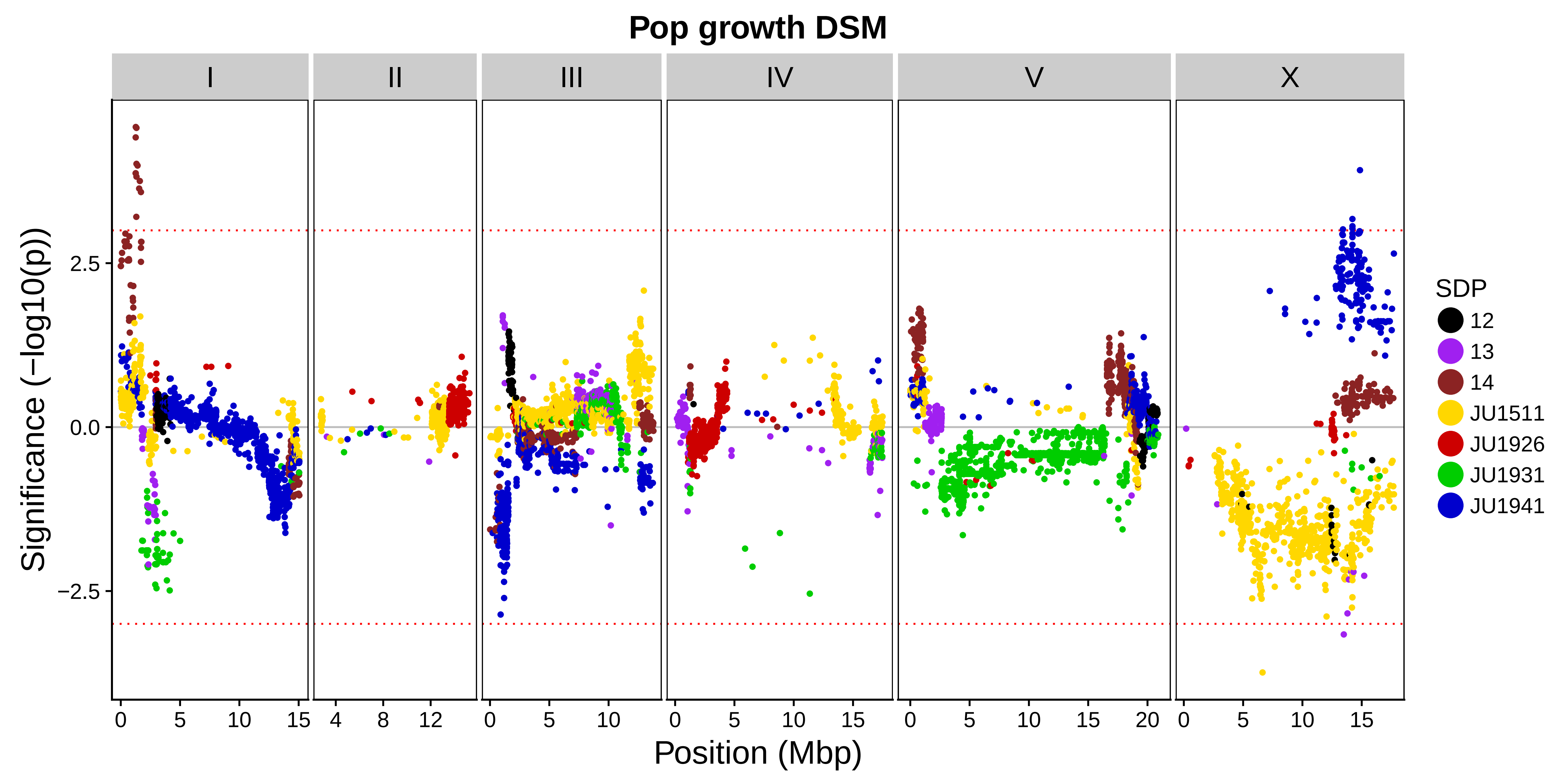

Pop growth DSM
I
II
III
IV
V
X
Significance (−log10(p))
2.5
0.0
−2.5
0
5
10
15
4
8
12
20
Position (Mbp)
SDP
12
13
14
JU1511
JU1926
JU1931
JU1941

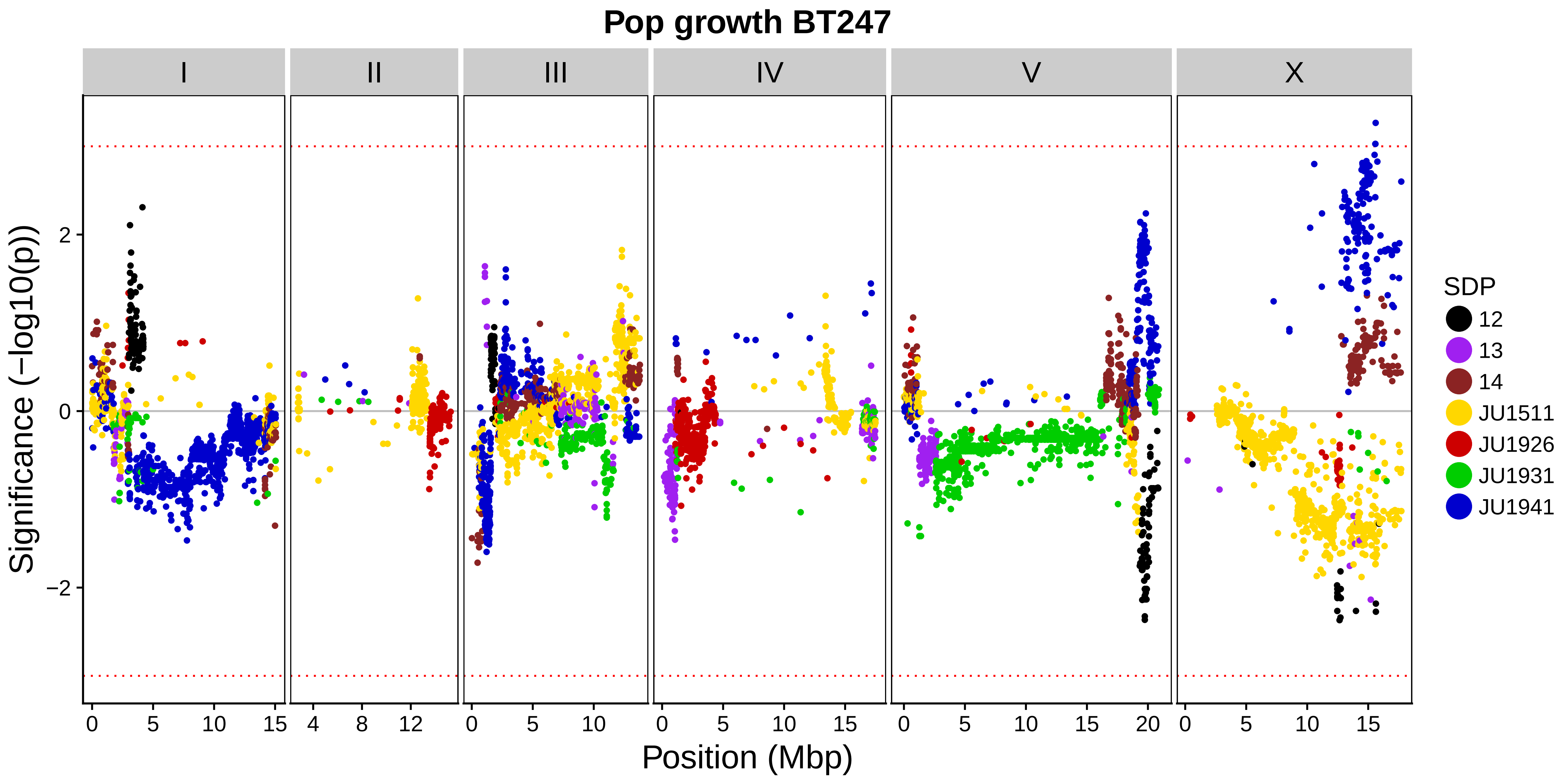

Pop growth BT247
I
II
III
IV
V
X
Significance (−log10(p))
2
0
−2
0
5
10
15
4
8
12
0
5
10
0
5
10
15
0
5
10
15
20
0
5
10
15
Position (Mbp)
SDP
12
13
14
JU1511
JU1926
JU1931
JU1941

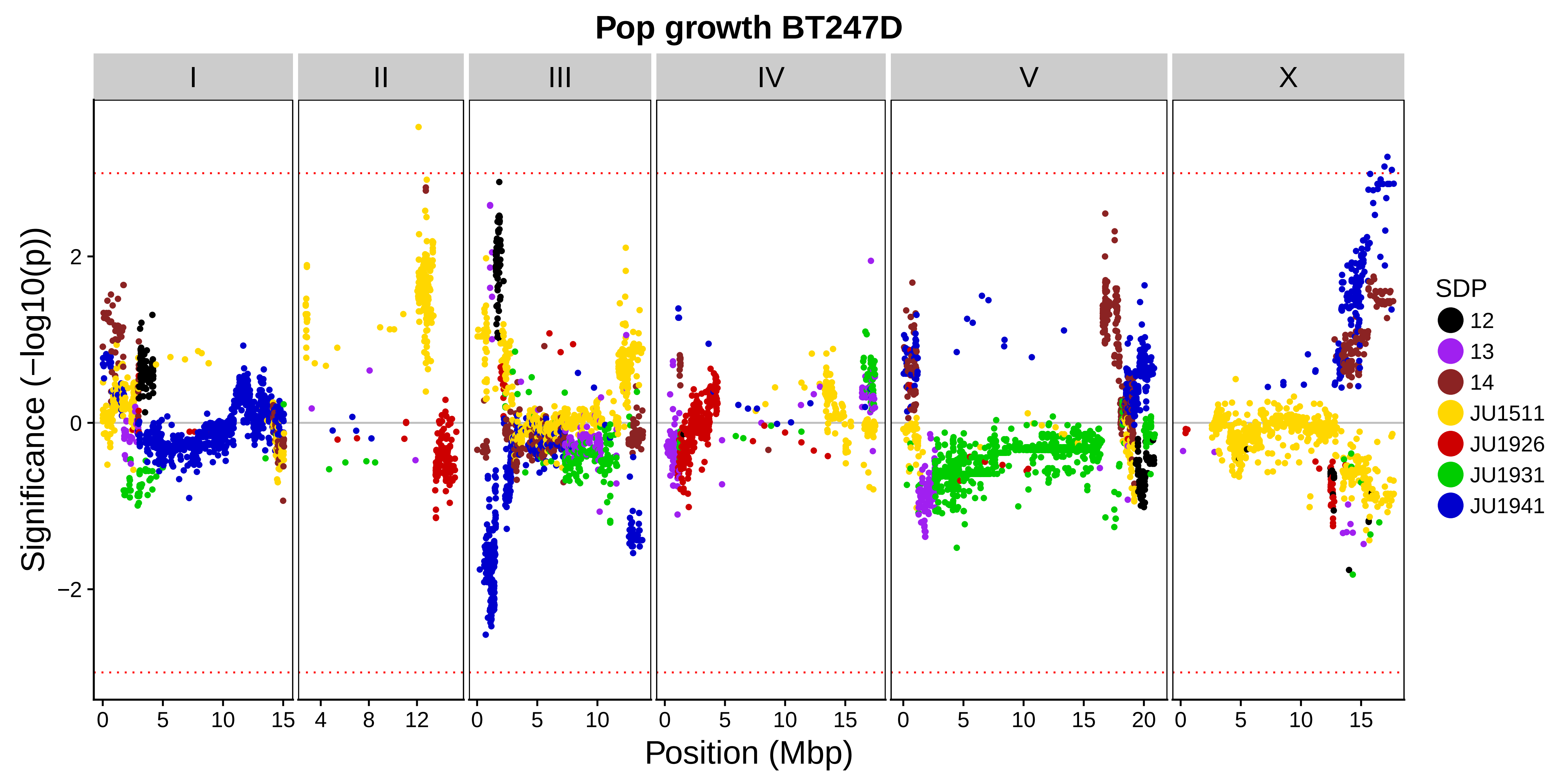

Pop growth BT247D
I
II
III
IV
V
X
Significance (−log10(p))
2
0
−2
0
5
10
15
4
8
12
0
5
10
0
5
10
15
0
5
10
15
20
0
5
10
15
Position (Mbp)
SDP
12
13
14
JU1511
JU1926
JU1931
JU1941

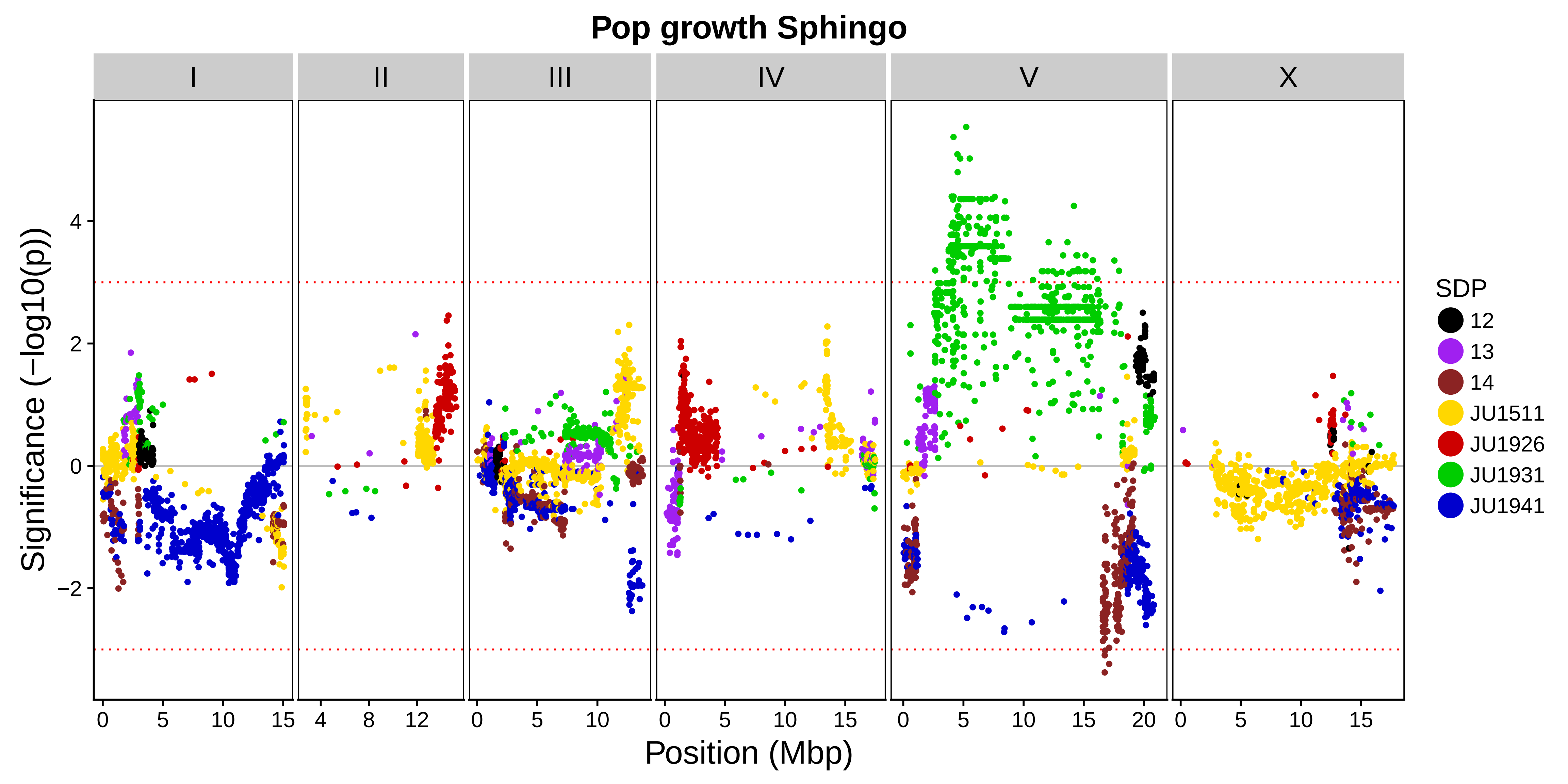

Pop growth Sphingo
I
II
III
IV
V
X
Significance (−log10(p))
4
2
0
−2
Position (Mbp)
0
5
10
15
4
8
12
20
SDP
12
13
14
JU1511
JU1926
JU1931
JU1941
